# Herpes simplex virus type 1 modifies the protein composition of extracellular vesicles to promote neurite outgrowth and neuroinfection

**DOI:** 10.1128/mbio.03308-23

**Published:** 2024-01-26

**Authors:** Guorong Sun, Kai Alexander Kropp, Marieluise Kirchner, Nina Plückebaum, Anton Selich, Manutea Serrero, Akshay Dhingra, Jorge Rubén Cabrera, Birgit Ritter, Rudolf Bauerfeind, Emanuel Wyler, Markus Landthaler, Axel Schambach, Beate Sodeik, Philipp Mertins, Abel Viejo-Borbolla

**Affiliations:** 1 Institute of Virology, Hannover Medical School, Hannover, Germany; 2 Proteomics platform, Max-Delbrück-Center for Molecular Medicine in the Helmholtz Association (MDC) and Berlin Institute of Health (BIH), Berlin, Germany; 3 Institute of Experimental Hematology, Hannover Medical School, Hannover, Germany; 4 Centro de Biología Molecular Severo Ochoa, Consejo Superior de Investigaciones Científicas—Universidad Autónoma de Madrid, Madrid, Spain; 5 Research Core Unit for Laser Microscopy, Hannover Medical School, Hannover, Germany; 6 Berlin Institute for Medical Systems Biology (BIMSB), Max Delbrück Center for Molecular Medicine in the Helmholtz Association, Berlin, Germany; 7 Institute for Biology, Humboldt University of Berlin, Berlin, Germany; 8 Cluster of Excellence-Resolving Infection Susceptibility (RESIST, EXC 2155), Hannover Medical School, Hannover, Germany; The University of North Carolina at Chapel Hill School of Medicine, Chapel Hill, North Carolina, USA; The University of North Carolina at Chapel Hill School of Medicine, Chapel Hill, North Carolina, USA

**Keywords:** herpes simplex virus, neurite outgrowth, neuroinfection, extracellular vesicles, galectin-1

## Abstract

The highly prevalent herpes simplex virus type 1 (HSV-1) causes a range of diseases, including cold sores, blinding keratitis, and life-threatening encephalitis. HSV-1 initially replicates in epithelial cells, enters the peripheral nervous system via neurites, and establishes lifelong infection in the neuronal cell bodies. Neurites are highly dynamic structures that grow or retract in response to attractive or repulsive cues, respectively. Here, we show that infection with HSV-1, but not with a mutant virus lacking glycoprotein G (gG), reduced the repulsive effect of epithelial cells on neurite outgrowth and facilitated HSV-1 invasion of neurons. HSV-1 gG was required and sufficient to induce neurite outgrowth by modifying the protein composition of extracellular vesicles, increasing the amount of neurotrophic and neuroprotective proteins, including galectin-1. Antibodies directed against galectin-1 neutralized the capacity of extracellular vesicles released from HSV-1-infected cells to promote neurite outgrowth. Our study provides new insights into the neurotropism of HSV-1 and identifies a viral protein that modifies the protein composition of extracellular vesicles to stimulate neurite outgrowth and invasion of the nervous system.

**IMPORTANCE:**

Herpes simplex virus type 1 (HSV-1) must infect neurites (or nerve endings) to establish a chronic infection in neurons. Neurites are highly dynamic structures that retract or grow in the presence of repulsive or attractive proteins. Some of these proteins are released by epithelial cells in extracellular vesicles and act upon interaction with their receptor present on neurites. We show here that HSV-1 infection of epithelial cells modulated their effect on neurites, increasing neurite growth. Mechanistically, HSV-1 glycoprotein G (gG) modifies the protein composition of extracellular vesicles released by epithelial cells, increasing the amount of attractive proteins that enhance neurite outgrowth and facilitate neuronal infection. These results could inform of therapeutic strategies to block HSV-1 induction of neurite outgrowth and, thereby, neuronal infection.

## INTRODUCTION

Herpes simplex virus type 1 and its close relative type 2 (HSV-1 and HSV-2) are widespread human pathogens, with estimated prevalences of 67% and 13%, respectively, in people under the age of 50 (1). Infection with HSV-1 and HSV-2 can be asymptomatic or cause a wide variety of diseases, including mild cold sores, blinding herpes stromal keratitis, and life-threatening encephalitis as well as disseminated disease in the neonate, affecting life quality and causing high morbidity, mortality, and economic losses (2, 3). Initial HSV infection occurs in epithelial cells of the orolabial and genital mucosa as well as in the skin and cornea (4). Following replication in epithelial cells, HSV-1 and HSV-2 reach and enter neurites to colonize neurons and establish lifelong latency in the ganglia of the peripheral nervous system (PNS) (5–8). Latent HSV-1 and HSV-2 reactivate frequently, producing infectious viruses that travel in an anterograde manner within neurites toward peripheral tissues, where they cause recurrent diseases and spread to other individuals (9, 10).

Neurites play key roles in HSV infection as well as transmission from peripheral tissue to ganglia and back. They are highly dynamic structures that grow or retract in the presence of attractive or repulsive cues, respectively, expressed by different cell types, including epithelial cells in the mucosa and skin (11, 12). Some of these cues can be released as secreted proteins or as part of extracellular vesicles (EVs). For instance, secreted proteins semaphorin 3A and nerve growth factor (NGF) inhibit and increase, respectively, neurite outgrowth (11, 13, 14). Galectin-1 located in EVs induces neurite outgrowth in several scenarios, including in adult tissue, through interaction with neuropilin-1/plexinA4 receptor complex (15–18). An example of a protein released in EVs that inhibits neurite outgrowth and regeneration is Nogo-A (19).

HSV-1 and HSV-2 have co-evolved with humans for millions of years and acquired specific strategies to establish lifelong infection of neurons. Upon reactivation from human sacral ganglia and infection of keratinocytes in the genital skin, HSV-2 increases expression of interleukin 17c (IL-17c), a cytokine that induces neurite outgrowth (20). Peng and colleagues suggested that the enhanced neurite outgrowth would protect neurons from nerve damage and potentially neuronal death that could occur following frequent HSV-2 reactivation (20). We previously showed that the purified, secreted domain of glycoprotein G (gG) from HSV-2, but not the ectodomain of HSV-1 gG, increases neurite outgrowth in an NGF-dependent manner (21). HSV-2 gG also enhances NGF-mediated neurite outgrowth during infection, by inhibiting the repulsion that some non-neuronal cells have on neurite outgrowth (22). HSV-1 and HSV-2 gG are the most divergent glycoproteins between these two viruses. The N-terminal domain of HSV-2 gG is secreted following cleavage by a furin-like protease, while HSV-1 gG is not cleaved during infection (23–26). Overall, these results clearly show that HSV-2 induces neurite outgrowth by modulating the activity and expression of neurotrophic factors.

HSV-1 is more prevalent than HSV-2 and causes encephalitis more frequently than HSV-2 (2, 3), suggesting better interindividual spread and more common infection of the brain. Moreover, there is the hypothesis that HSV-1 recurrent infection increases the risk of neurodegenerative diseases (for a review see Laval and Enquist, 2021 (27)). HSV-1 gG plays relevant roles in HSV-1 entry. HSV-1 lacking gG can efficiently infect cells through the endocytic pathway (28), while another study showed that HSV-1 gG is important for efficient infection through the apical surface of polarized epithelial cells, such as those encountered by the virus during primary infection (29). Moreover, three reports showed that HSV-1 strains lacking gG expression are attenuated *in vivo*, showing also less infectivity in the nervous system, by unknown mechanisms (30–32). Whether this is related to a potential role of gG in the induction of neurite outgrowth, facilitating neuronal infection is unknown. Our previous work addressing the role of HSV gG in neurite outgrowth was performed with recombinant purified gG ectodomain (21). However, our unpublished data performed with epithelial cells transfected with full-length gG suggested that this protein could modulate neurite outgrowth through a different mechanism than HSV-2 gG. Here, we addressed this question employing well-characterized neurite outgrowth repulsion models using conditioned medium from epithelial cells and co-culture systems (21, 22, 33, 34). Expression of full-length HSV-1 gG in epithelial cells, alone or in the context of infection, reduced their repulsive effect on neurite outgrowth, facilitating HSV-1 spread to neurons. Mechanistically, HSV-1 gG modified the protein composition of EVs, leading to increased levels of several proteins with neuronal activities, including galectin-1. Overall, our results show a novel strategy employed by HSV-1 to increase neurite outgrowth and HSV-1 spread to neurons.

## RESULTS

### HSV-1 gG inhibits the repulsion of epithelial cells on neurite outgrowth

**Fig 1:**
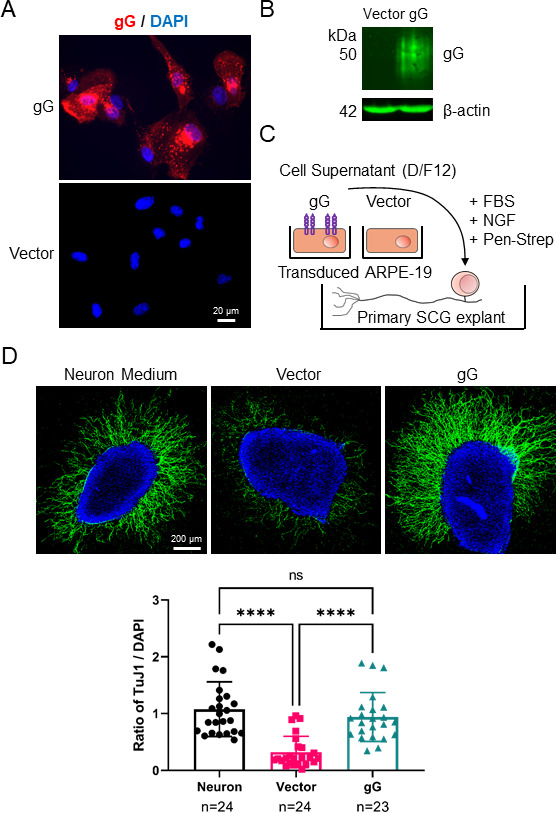
Expression of HSV-1 gG inhibits the repulsion of epithelial cells on neurite outgrowth. (**A**) Immunofluorescence microscopy images showing expression of HSV-1 gG (red) in stably transduced ARPE-19 cells. Nuclei were stained with DAPI (blue). Scale bar: 20 µm. (**B**) Immunoblot detecting HSV-1 gG and β-actin in cell lysates of stably transduced ARPE-19 cells. (**C**) Schematic representation of the neurite outgrowth assay. SCG from neonatal mice were seeded on collagen matrix and cultured with commercial neuron medium or conditioned medium from vector- or HSV-1 gG-transduced ARPE-19 cells supplemented with serum, NGF, and antibiotics. (**D**) The top panels show representative confocal microscopy images of SCG incubated with neuron medium or conditioned medium from stably transduced ARPE-19 cells. After 20–24 hours, SCGs were fixed and labeled with anti-β-III-tubulin antibody (TuJ1, green) and stained with DAPI (blue). Scale bar: 200 µm. The graph below shows the quantification of neurite outgrowth in each experimental group, presented as the ratio of neurite fluorescence intensity (TuJ1) to DAPI signal intensity for each SCG. Quantification was performed with FIJI software (see Materials and Methods). Each symbol corresponds to one SCG. N indicates the number of SCG in each experimental condition. Data are presented as mean ± standard deviation of the mean. ns, not significant; **** *P* < 0.0001 (Kruskal-Wallis test with Dunn’s multiple comparisons test).

Neurites are key for neuronal colonization by HSV-1, and they are therefore likely modulated by this virus. The secreted domain of HSV-2 gG enhances NGF-dependent neurite outgrowth, while the ectodomain of HSV-1 gG does not (21). Here, we investigated the impact of full-length HSV-1 gG on neurite outgrowth employing ARPE-19 retinal pigment epithelial cells, whose conditioned medium inhibits neurite outgrowth (22). We generated stably transduced ARPE-19 cells constitutively expressing full-length HSV-1 gG and confirmed its expression by immunofluorescence microscopy and immunoblot (Fig. 1A and B). Next, we cultured mouse superior cervical ganglia (SCG) of neonatal mice in a collagen matrix in the presence of regular neuron medium, or of conditioned medium from transduced ARPE-19 cells, and quantified neurite outgrowth 20–24 hours later (Fig. 1C). All conditioned media were supplemented with NGF that is required for survival of SCG neurons and induces neurite outgrowth. The presence of conditioned medium from vector-only transduced cells reduced neurite outgrowth compared to the normal neuronal medium control. However, the expression of HSV-1 gG inhibited the repulsion, resulting in similar neurite outgrowth as in the ganglia exposed to the normal neuronal medium (Fig. 1D). We obtained similar results when experimenting with conditioned medium from vector- or gG-transfected HEK293-T cells (Fig. S1). These results show that ectopic expression of full-length HSV-1 gG reverts the repulsion on neurite outgrowth exerted by ARPE-19 and HEK293-T cells.

### HSV-1 gG expressed during infection inhibits epithelial cell repulsion on neurite outgrowth

**Fig 2:**
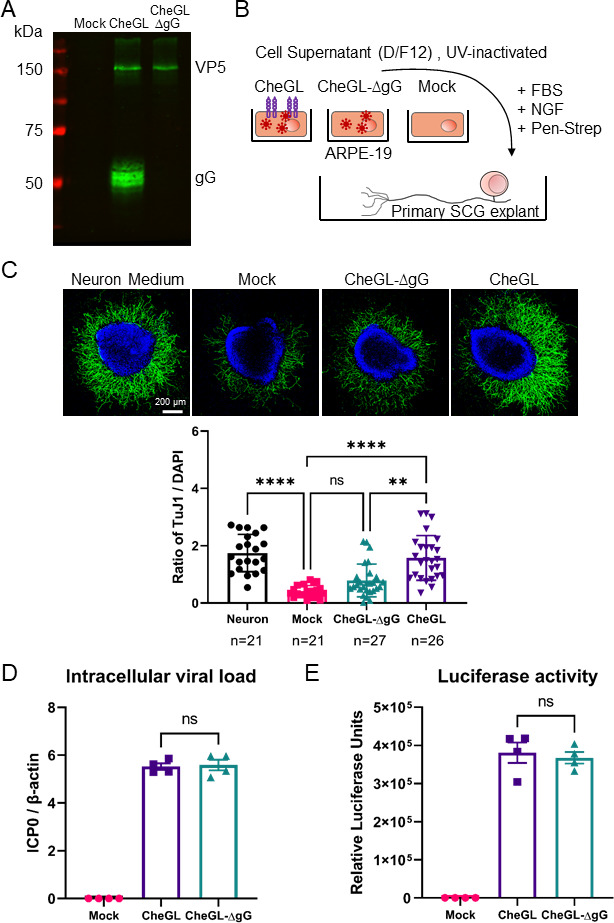
HSV1-CheGL, but not HSV1-CheGL-ΔgG, inhibits the repulsion of epithelial cells on neurite outgrowth. (**A**) Immunoblot detecting HSV-1 gG and major capsid protein VP5 in cell lysates of infected ARPE-19 cells. (**B**) Schematic representation of the neurite outgrowth assay. SCG from neonatal mice were seeded on collagen matrix and cultured with commercial neuron medium or conditioned medium from mock- or HSV-1-infected ARPE-19 cells supplemented with serum, NGF, and antibiotics. (**C**) The top panels show representative immunofluorescence confocal images of SCG incubated with neuronal medium or conditioned medium from mock- or HSV-1-infected ARPE-19 cells. The media were treated with ultraviolet light (UV) to inactivate HSV-1. After 20–24 hours, SCGs were fixed and labeled with anti-β-III-tubulin antibody (TuJ1, green) and stained with DAPI (blue). Scale bar: 200 µm. The graph below shows the quantification of neurite outgrowth in each experimental group, presented as the ratio of neurite fluorescence intensity (TuJ1) to DAPI signal intensity for each SCG. Quantification was performed with FIJI software (see Materials and Methods). Each symbol corresponds to one SCG. N indicates the number of SCG in each experimental condition. (**D**) Graph showing intracellular viral genome copy numbers per cellular genome, detected by qPCR in DNA obtained from mock- and HSV-1-infected ARPE-19 cells at the time of supernatant collection (16 hours post-infection). HSV-1 genome copy number was quantified by amplifying ICP0, while the host genome was quantified by amplifying β-actin. Genome copy numbers were calculated by generating standard curves and presented as a ratio of ICP0 to β-actin. (**E**) Graph showing the amount of *Gaussia* luciferase detected in the supernatant of infected ARPE-19 cells at the time of supernatant collection (16 hpi). Data are presented as mean ± standard deviation of the mean. Ns, not significant; ** *P* < 0.01, ****P < 0.0001 (Kruskal-Wallis test with Dunn’s multiple comparisons test).

To investigate whether HSV-1 gG modulated neurite outgrowth during infection, we first generated an HSV-1 reporter virus lacking gG expression by *en-passant* mutagenesis (35, 36) (Fig. S2A). To do so, we employed HSV1-CheGL derived from the clinical isolate HSV-1 17^+^, cloned into a bacterial artificial chromosome, and expressing monomeric Cherry (Che) and *Gaussia* luciferase (GL) separated by a picornavirus 2A site under the control of the major immediate early promoter of human cytomegalovirus (37). The resulting virus was termed HSV1-CheGL-ΔgG (see Materials and Methods). Lack of gG expression in HSV1-CheGL-ΔgG was confirmed by immunoblot (Fig. 2A) and did not affect HSV-1 replication in Vero and ARPE-19 cells (Fig. S2B), in line with previous reports in which HSV-1 strains lacking gG expression were not impaired *in vitro* (30–32).

Next, we determined whether HSV-1 infection affected ARPE-19 neurite repulsion (Fig. 2B and C). To do so, we collected conditioned media at 16 hours post-infection (hpi), and inactivated the virus with ultraviolet (UV) light, prior to incubation with SCG (see Materials and Methods). Conditioned media from mock- or HSV1-CheGL-ΔgG-infected ARPE-19 cells reduced neurite outgrowth from SCG while that from ARPE-19 cells infected with HSV1-CheGL did not (Fig. 2C). Cells infected with HSV1-CheGL or HSV1-CheGL-ΔgG contained similar levels of viral genomes and secreted similar amounts of luciferase, demonstrating that the lower neurite outgrowth was not due to a reduced infection with HSV1-CheGL-ΔgG than with HSV1-CheGL (Fig. 2D and E). We obtained similar results when experimenting with conditioned medium from mock-, HSV1-CheGL-, and HSV1-CheGL-ΔgG-infected HEK293-T cells (Fig. S3). Taken together, these results indicated that gG expressed during HSV-1 infection reduced the repulsion of ARPE-19 and HEK293-T cells, leading to higher neurite outgrowth.

### HSV-1 gG stimulation of neurite outgrowth promotes neuronal infection

**Fig 3:**
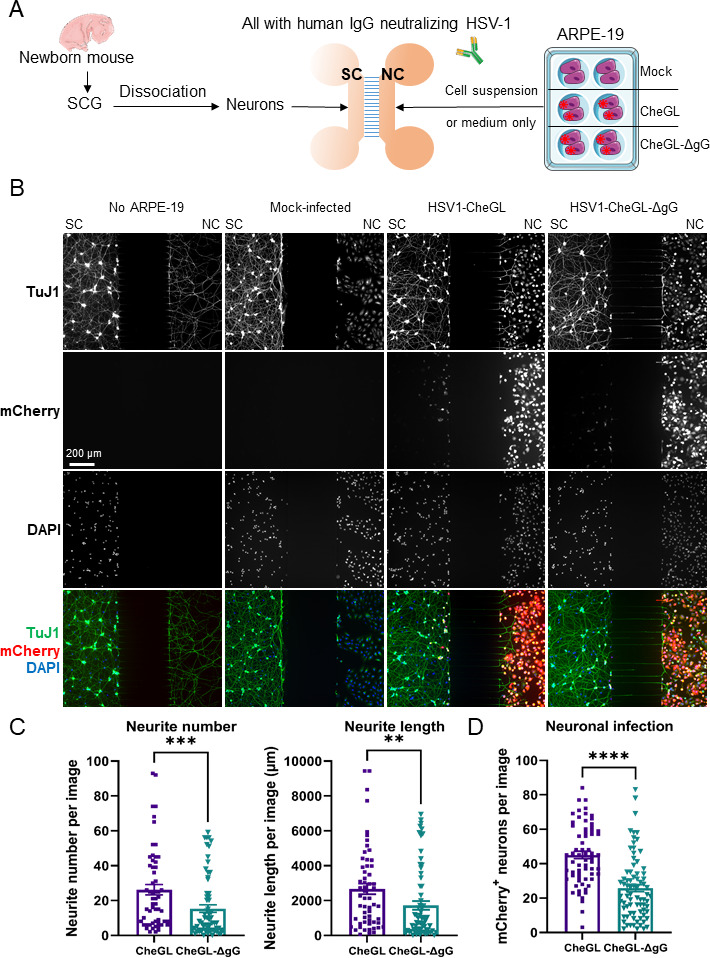
HSV1-CheGL induces more neurite outgrowth than HSV1-CheGL-ΔgG toward infected ARPE-19 cells, facilitating neuronal infection. (**A**) Schematic representation of the experiment performed to quantify neurite outgrowth and neuronal infection using MFC. SCGs were extracted from neonatal mice and dissociated into single neurons that were seeded into the soma compartment (SC) of MFC. Mock- or HSV-1-infected ARPE-19 cells were added into the neurite compartment (NC) simultaneously. The ARPE-19 cells had been infected with HSV-1 at an MOI of 1 for 16 hours prior to seeding into the NC. The darker color of the NC than the SC indicates that the NC contained more volume to facilitate the diffusion of factors secreted by ARPE-19 cells into the SC. To avoid diffusion of cell-free HSV-1 from infected ARPE-19 cells, we added anti-HSV-1 neutralizing antibodies. (**B**) Immunofluorescence microscopy images of neurons and ARPE-19 cells grown in MFC. Dissociated SCG neurons were seeded in the SC in all experimental conditions. The absence or presence of mock-, HSV1-CheGL-, or HSV1-CheGL-ΔgG-infected ARPE-19 cells in the NC is indicated above each column. After 20–24 hours, the cells were fixed and labeled with an anti-β-III-tubulin antibody (TuJ1, top row). HSV-1-infected cells were detected by mCherry expression (second row) and nuclei were detected with DAPI staining (third row). These panels show the gray channels, while the bottom row shows images containing all detected signals in their respective colors (TuJ1, green; mCherry, red; DAPI, blue). Scale bar: 200 µm. (**C and D**) Graphs showing total neurite number and length in the NC of MFCs (**C**) and number of mCherry positive neurons in the SC (**D**). Quantification was performed with FIJI software (see Materials and Methods). Each symbol represents the data from one picture taken randomly and covers both sides next to the microgrooves in the MFC. Data are presented as mean ± standard error of the mean. ** *P* < 0.01, ***P < 0.001, ****P < 0.0001 (Mann-Whitney test). Abbreviations: SCG, superior cervical ganglia; IgG, immunoglobulin; SC, somal compartment; NC, neurite compartment.

To address whether the increased neurite outgrowth mediated by HSV-1 gG could impact the infection of peripheral neurons, we performed experiments with microfluidic chambers (MFC) that separate neuronal cell bodies and neurites (22, 33, 34, 38–40). These devices allow selective infection via the neurites or the somata, permitting mechanistic experiments that cannot be performed *in vivo*. We seeded dissociated SCG neurons of newborn mice in the soma compartment (SC) of MFC and added simultaneously into the neurite compartment (NC) mock-, HSV1-CheGL-, or HSV1-CheGL-ΔgG-infected ARPE-19 cells (Fig. 3A). The ARPE-19 cells had been infected for 16 hours with an MOI of 1 prior to seeding into NC. We added more volume in the NC than in the SC to permit the factors released by epithelial cells to diffuse into the SC (see Materials and Methods). We also applied neutralizing immunoglobulins to the whole MFC to ensure that HSV-1 infection of neurons could only occur via cell-to-cell spread and not via released extracellular HSV-1 particles (41) that diffused into the SC. We showed the efficiency of the immunoglobulins by seeding non-infected ARPE-19 cells in the SC and HSV1-CheGL- or HSV1-CheGLΔgG-infected ARPE-19 in the NC in the absence or presence of HSV-1 neutralizing antibodies (Fig. S4). The lack of neutralizing antibodies allowed diffusion of HSV-1 from the NC to the SC, while the presence of the antibodies inhibited virion diffusion and thereby infection of ARPE-19 cells in the SC (Fig. S4).

After 20–24 hours of co-culture in the MFC, we collected the supernatant from the NC and measured luciferase activity, as a surrogate of HSV-1 infection, obtaining similar levels for ARPE-19 cells infected with HSV1-CheGL and HSV1-CheGLΔgG (Fig. S5). Next, we fixed the cells and labeled them with anti-β-III-tubulin and quantified neurite number and neurite length at the NC side. While the neurons extended many β-III-tubulin-positive neurites from the SCs to the NC lacking ARPE-19 cells, the presence of mock-infected ARPE-19 cells in the NC almost completely inhibited neurite outgrowth from the SC toward the NC (Fig. 3B, top panels). By contrast, infection of ARPE-19 cells with HSV1-CheGL increased neurite number and length more than infection with HSV1-CheGL-ΔgG (Fig. 3B and C). These results confirm previous experiments with conditioned media from infected ARPE-19 and HEK293-T cells (Fig. 2C; Fig. S1B, respectively), indicating that HSV-1 infection overcame their repulsion on neurite outgrowth and that gG was required for this phenotype.

To determine whether the increased neurite outgrowth facilitated infection of neurons, we quantified the number of mCherry-positive neuronal cell bodies in the SC. Infection of ARPE-19 cells with HSV1-CheGL led to a higher number of mCherry-positive neurons in the SC than infection with HSV1-CheGL-ΔgG (Fig. 3B and D). To eliminate the possibility that the reduced number of mCherry-positive cell bodies observed with HSV1-CheGL-ΔgG could be due to an impairment of this virus to infect neurons in the MFC, we performed experiments in the absence of neutralizing antibodies and obtained a similar number of mCherry positive neurons in the SC, irrespective of the virus employed to infect ARPE-19 cells (Fig. S6). In another experiment, we first seeded the neurons into the SCs for 24 hours to allow similar neurite extension through the microgrooves into the NCs, prior to the addition of infected ARPE-19 cells in the presence of neutralizing antibodies. This allowed efficient contact between neurites and infected ARPE-19 cells. In this condition, HSV1-CheGL and HSV1-CheGL-ΔgG infected the neurons to a comparable extent (Fig. S7). These data indicated that the neurons were equally susceptible to both viruses, if provided equal access and that gG was not required for efficient HSV-1 entry into neurons nor subsequent events leading to reporter gene expression. Overall, these results suggested that HSV-1 reduced the repulsion of epithelial cells, increasing neurite outgrowth and facilitating spread to neurons, but neurite outgrowth and neuronal infection were less pronounced in the absence of HSV-1 gG.

### HSV-1 gG increases neurite outgrowth *via* modification of extracellular vesicles

**Fig 4:**
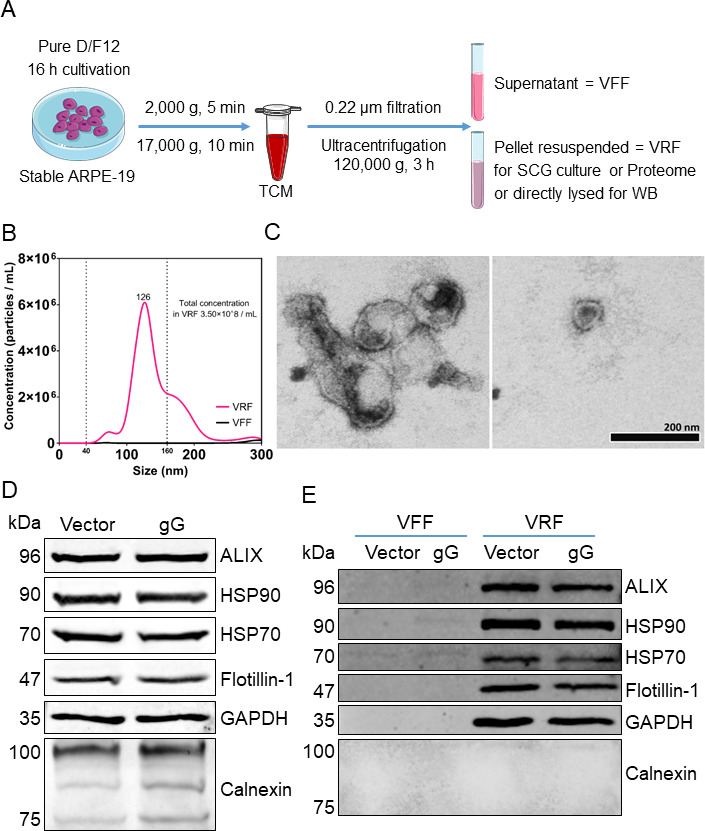
Fractionation of conditioned medium from ARPE-19 cells into vesicle-free and vesicle-rich fractions. (**A**) Schematic diagram showing the fractionation strategy to separate the conditioned medium of stably transduced ARPE-19 cells into VFF and VRF. (**B**) Graph showing the concentration and size distribution of EV in VRF and VFF (pink and black curves, respectively) determined with a NanoSight LM10 instrument. (**C**) Representative electron micrograph of the VRF following negative staining. Scale bar: 200 nm. (**D and E**) Immunoblot detecting several EV markers in whole-cell lysates (**D**) as well as in VFF and VRF (**E**) from the conditioned medium of stably transduced ARPE-19 cells.

The conditioned medium of cultured cells contains mainly secreted factors and extracellular vesicles (EVs). To check which of these two fractions of the conditioned medium plays a role in neurite outgrowth in our experimental model, we separated them by filtration and centrifugation, using an established protocol (42) (Fig. 4A; see Materials and Methods). We obtained a fraction abundant in vesicles, termed vesicle-rich fraction (VRF), and a vesicle-depleted fraction, termed vesicle-free fraction (VFF). Nanoparticle analysis showed that the vesicles of the VRF had a diameter of 50–200 nm with a peak around 125 nm (Fig. 4B, pink curve), consistent with other reports on EVs (42, 43), while very few vesicles were detected in the VFF (Fig. 4B, black curve). We also observed EVs in the VRF using transmission electron microscopy (Fig. 4C). Next, we further characterized the VFF and VRFs by immunoblot with a set of general EV markers (44). ALG-2-interacting protein X (Alix), heat shock protein 70 (HSP70), HSP90, flotillin-1, and glyceraldehyde-3-phosphate dehydrogenase (GAPDH) were present in whole-cell lysates (Fig. 4D) and had fractionated almost exclusively in the VRF but not in the VFF (Fig. 4E). Moreover, calnexin, a widely used exclusion marker of EVs (43, 44), was only detected in whole-cell lysates but not in the VFF and VRF (Fig. 4D and E).

**Fig 5:**
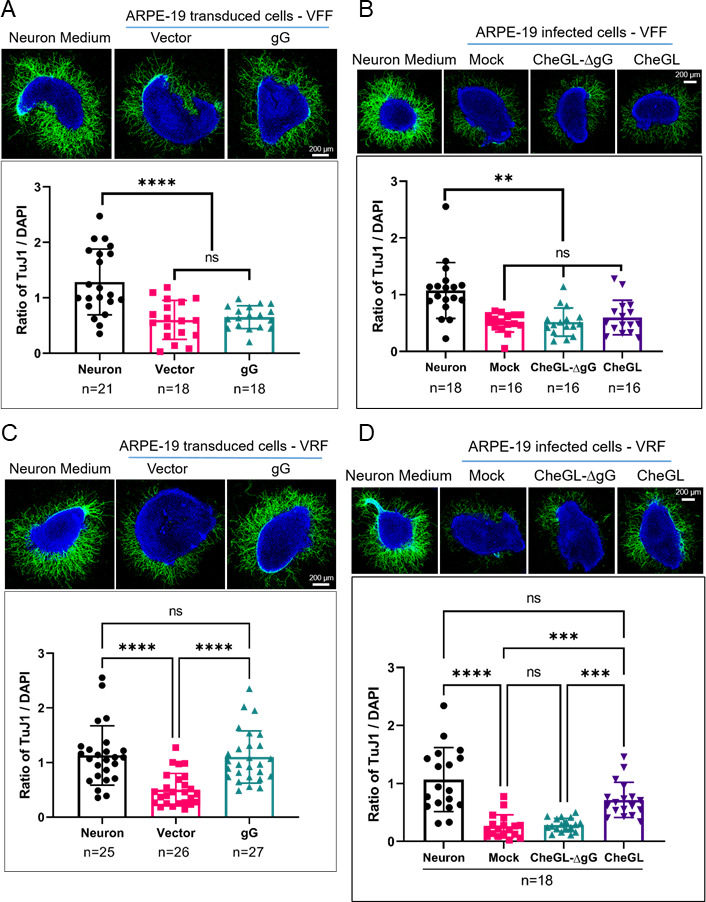
HSV-1 gG inhibits the repulsion of VRF, but not of VFF, on neurite outgrowth. The top panels show representative immunofluorescence confocal images of SCG incubated with normal neuron medium or VFF/VRF from stably transduced (**A and C**) or infected (**B and D**) ARPE-19 cells. The neurites were labeled with TuJ1 (green) and the nuclei were stained with DAPI (blue). Scale bar: 200 µm. The graphs show the ratio of neurite fluorescence intensity (TuJ1) to DAPI signal intensity. Quantification was performed with FIJI software (see Materials and Methods). Each symbol represents one SCG. Data are presented as mean ± standard deviation of the mean. N indicates the number of SCG in each experimental condition. Abbreviations: VFF, vesicle-free fraction; VRF, vesicle-rich fraction; ns, not significant; ** *P* < 0.01; ***P < 0.001; ****P < 0.0001 (Kruskal-Wallis test with Dunn’s multiple comparisons test).

We then performed neurite outgrowth assays by incubating SCG explants with VFF or VRF obtained from stably transduced or infected ARPE-19 cells. We found that VFF isolated from stably transduced and infected ARPE-19 cells inhibited neurite outgrowth regardless of gG expression (Fig. 5A and B). By contrast, VRF derived from vector-transduced cells repelled neurite outgrowth, while the repulsion was reduced with VRF from gG-expressing ARPE-19 cells (Fig. 5C). Similarly, VRF obtained from ARPE-19 cells infected with HSV1-CheGL-ΔgG or mock-treated inhibited neurite outgrowth, while VRF from ARPE-19 cells infected with the parental HSV1-CheGL reverted this phenotype (Fig. 5D).

The VRF from infected cells also contains virions (Fig. S8A). To determine whether the virions induce neurite outgrowth, we purified them using multiple gradient ultracentrifugation. The purified bands contained viral proteins but not the EV marker GAPDH (Fig. S8B). We inactivated the virions by UV, resuspended them in neuron media, and added them to SCG explants. We did not detect significant differences in neurite outgrowth after 20-h incubation (Fig. S8C and D).

These results suggest that HSV-1 gG increased neurite outgrowth by modulating factors present in the VRF but not in the VFF, and this function was most likely mediated by EV components, not virions.

### Expression of HSV-1 gG regulates the protein composition of EVs

To determine the protein composition of VRF, we performed liquid chromatography-mass spectrometry (LC-MS/MS) from VRF obtained from vector- and HSV-1 gG-transduced ARPE-19 cells. Of the 214 proteins included for quantitation, 178 have been previously annotated as proteins present in the extracellular milieu (source: http://annotations.perseus-framework.org/; 2020). The FDR (false discovery rate) was set to 1% for peptide and protein identifications. We considered unique and razor peptides for quantification. Five replicates were assigned to each experimental group and data were filtered for a minimum value of three in at least one group, and for proteins with a minimum of three MS/MS counts. Differential protein abundance was calculated using Student’s *t*-test. Top-regulated proteins were defined with a cutoff of 15% FDR. We included the normalized quantitative protein data from our mass spectrometry-based analyses in Table S1.

**Fig 6:**
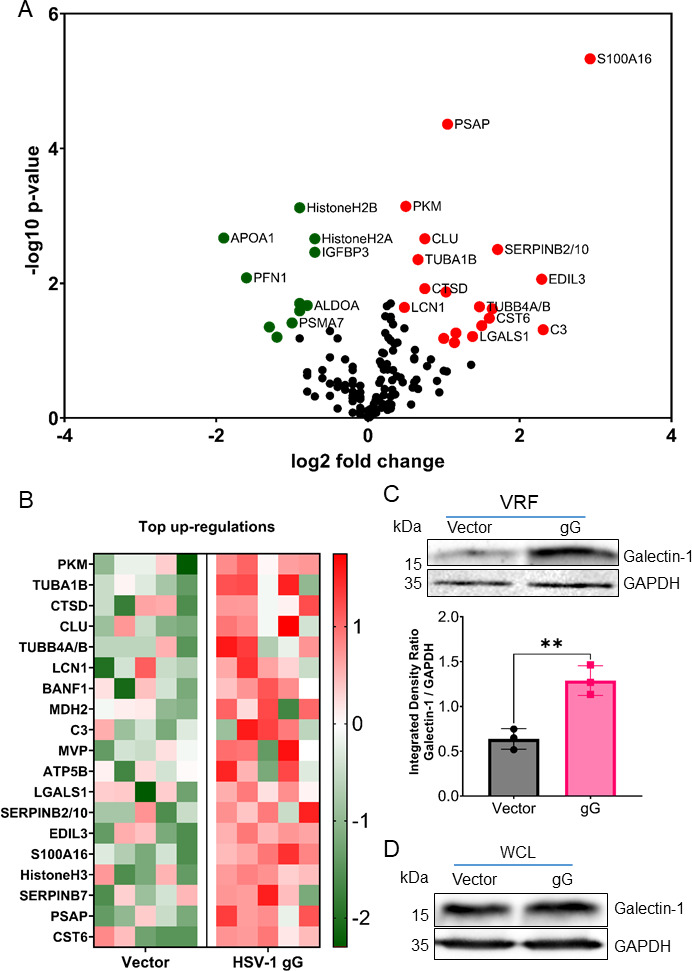
HSV-1 gG modifies the protein composition of EV. Liquid chromatography-mass spectrometry was performed on VRF obtained from vector- or gG-transduced ARPE-19 cells (five biological replicates per condition). (**A**) Volcano plot displaying up-regulated (red) and down-regulated (green) proteins determined using student´s *t*-test with a significance cutoff of 15% FDR. (**B**) Heatmap showing the names of genes whose protein products were increased in VRF of gG-expressing ARPE-19 cells compared to vector-transduced cells. The color code represents the raw z-scores. (**C and D**) Immunoblots detecting galectin-1 and GAPDH in VRF (**C**) and whole-cell lysates (WCL) (**D**) obtained from stably transduced ARPE-19. Data are presented as mean ± standard deviation of the mean. ** *P* < 0.01 (unpaired *t*-test with Welch’s correction)

The expression of HSV-1 gG resulted in the differential abundance of 30 proteins in VRF, with 19 being enriched and 11 being depleted due to HSV-1 gG expression (Fig. 6A and B). We did not detect HSV-1 gG in the VRF obtained from gG-transduced cells. Among the enriched proteins, several belong to families of proteins involved in neurite outgrowth, neurodegeneration, and neuronal activity. This includes S100A16, serpinB2/10, cathepsin D, and galectin-1 (16, 45–50). We focused on galectin-1 (*LGALS1*), which is neuroprotective and neurotrophic when present in EVs (15–17), and therefore might contribute to neurite outgrowth during HSV-1 infection. We confirmed the increase of galectin-1 in the VRF derived from gG-expressing cells, while galectin-1 level was not significantly changed in the whole-cell lysate (Fig. 6C and D), suggesting that HSV-1 gG mainly modulates its sorting into EVs but not its overall expression. These results indicated that the presence of HSV-1 gG modified the protein composition of EVs, including higher levels of galectin-1.

### EV-associated galectin-1 released by ARPE-19 cells enhances neurite outgrowth

**Fig 7:**
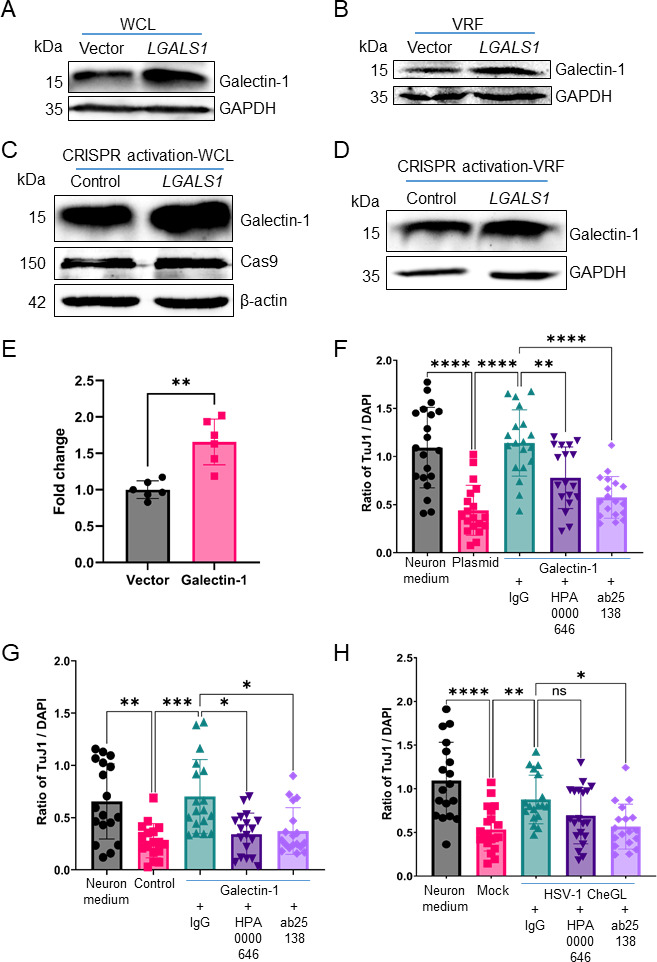
Galectin-1 promotes neurite outgrowth and this activity can be partially neutralized by antibodies. (**A and B**) Immunoblots detecting galectin-1 and GAPDH in whole-cell lysates (**A**) and VRF (**B**) of plasmid- or *LGALS1*-transduced ARPE-19 cells. (**C and D**) Immunoblots showing galectin-1, Cas9, and β-actin in whole-cell lysates (**C**) and galectin-1 and GAPDH in VRF (**D**) of ARPE-19 transfected with CRISPRa-*LGALS1* and CRISPRa-Control. (**E**) Quantification of Galectin-1 on non-permeabilized EV by flow cytometry. The graph shows the fold change of surface galectin-1-positive EVs derived from galectin-1 overexpressing ARPE-19 cells compared to vector-transduced ARPE-19 cells. Each symbol represents one sample from three independent experiments performed in duplicate. (**F**) Graph showing neurite outgrowth, represented as a ratio of TuJ1/DAPI signal, from SCG ganglia incubated during 20–24 hours with VRF obtained from plasmid- or *LGALS1*-transduced ARPE-19 cells in the presence of anti-galectin-1 neutralizing antibodies (HPA0000646 and ab25138) or IgG isotype control. SCGs were fixed and labeled with anti-β-III-tubulin antibody (TuJ1) and stained with DAPI. (**G and H**) Graphs showing neurite outgrowth, represented as ratio of TuJ1/DAPI signal, from SCG ganglia incubated during 20–24 hours with VRF obtained from ARPE-19 transfected with CRISPRa-*LGALS1* and CRISPRa-Control (**G**) or from HSV1-CheGL-infected ARPE-19 cells (**H**) in the presence of anti-galectin-1 neutralizing antibodies (HPA0000646 and ab25138) or IgG isotype control. SCGs were fixed and labeled with anti-β-III-tubulin antibody (TuJ1) and stained with DAPI. Data in E-H are presented as mean ± standard deviation of the mean. Abbreviations: WCL, whole-cell lysate; VRF, vesicle-rich fraction; ns, not significant; *P* < 0.05, **P < 0.01, ***P < 0.001, ****P < 0.0001 (Kruskal-Wallis test with Dunn’s multiple comparisons test).

To test whether EVs enriched for galectin-1 could overcome repulsion and restore neurite outgrowth, we generated ARPE-19 cells stably expressing galectin-1 (Fig. 7A and B), or transfected ARPE-19 cells with *LGALS1*-CRISPRa to increase *LGALS1* expression by clustered regularly interspaced short palindromic repeats activation (CRISPRa) (51, 52); (Fig. 7C and D). The cells generated with both approaches expressed more galectin-1 (Fig. 7A and C) and released VRFs containing more galectin-1 than those of their respective control cells, transduced or transfected with the corresponding control plasmids (Fig. 7B and D). A previous report showed the presence of galectin-1 on the surface of EV (15). Therefore, we addressed whether we could detect galectin-1 on non-permeabilized EV by flow cytometry. Our results suggest that a small percentage of EV contain galectin-1 on the surface of EVs. Moreover, they demonstrated an increase in the percentage of EVs containing galectin-1 on their surface in EVs derived from galectin-1 overexpressing cells (Fig. 7E; Fig. S9).

We then examined the effect of VRFs from different cellular conditions on neurite outgrowth. SCGs incubated with VRFs from galectin-1-expressing cells (Fig. 7F and G; green bars) projected more neurites than those incubated with the VRFs of the respective control cells (Fig. 7F and G; red bars). Furthermore, antibodies directed against galectin-1, namely IgG fraction ab25138 (Fig. 7F and G; light violet bars) with a documented neutralizing activity (15) or IgG fraction HPA0000646 (Fig. 7F and G; dark violet bars), diminished the VRF activity to overcome the repulsion and to stimulate neurite outgrowth, while an unspecific IgG fraction had not such an effect (Fig. 7F and G; green bars), irrespective of whether the VRFs were from ARPE-19 cells stably expressing galectin-1 (Fig. 7B) or from ARPE-19 cells stimulated for galectin-1 expression by CRISPRa (Fig. 7D).

We also addressed with these antibodies whether galectin-1 contributed to the HSV-1-induced promotion of neurite outgrowth. Incubation of SCGs with VRFs from mock-infected ARPE-19 cells repressed neurite outgrowth (Fig. 7H; red bar), while VRFs from HSV1-CheGL-infected ARPE-19 cells reduced the repulsion and restored neurite outgrowth also in the presence of the unspecific IgG fraction (Fig. 7H; green bars). By contrast, the anti-galectin-1 IgG fraction ab25138 (Fig. 7H; light violet bars) or IgG fraction HPA0000646 (Fig. 7H; dark violet bars) diminished the VRF activity to overcome the repulsion and to stimulate neurite outgrowth, although this effect only reached significance for the first antibody. Altogether, these data showed that EV-associated galectin-1 promoted neurite outgrowth and was partially responsible for HSV-1-mediated increase in neurite outgrowth. However, we cannot conclude that galectin-1 is the sole protein involved in this phenotype. The role in neurite outgrowth of other proteins enriched in EV upon gG expression deserves further investigation.

## DISCUSSION

**Fig 8:**
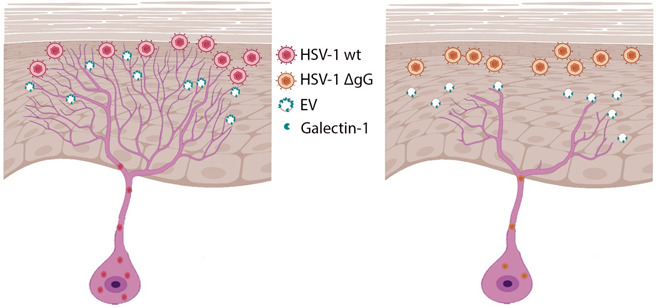
Model showing the potential effect of HSV-1 gG during infection. Infection of epithelial cells with HSV-1 (WT, left) or HSV-1 lacking gG expression (ΔgG, right) in the skin leads to the release of EVs. The EVs released by epithelial cells infected with HSV-1 WT contain higher levels of galectin-1 than those released upon HSV-1-ΔgG infection. Galectin-1 on EV induces neurite outgrowth, facilitating neuronal infection. Part of this figure was generated with Biorender.

Following the infection of epithelial cells, HSV-1 must enter into neurites to establish productive or latent infection in neurons. Epithelial cells release proteins that affect neurite outgrowth. Here, we show that HSV-1 infection inhibited the repulsion of epithelial cells on neurite outgrowth, leading to increased neurite length and better spread to neurons. Mechanistically, HSV-1 gG modifies the protein composition of EV. Among the proteins enriched in EV upon HSV-1 infection, we studied the role of galectin-1 and showed that this protein participated in the observed enhancement of neurite outgrowth (Fig. 8).

The closest relative of HSV-1, HSV-2, increases neurite outgrowth through several mechanisms. Following HSV-2 reactivation in sacral ganglia and infection of keratinocytes, infected cell protein 0 (ICP0) induces the expression of IL-17c, which stimulates neurite outgrowth in the human genital skin (20). Another mechanism involves the interaction between the secreted N-terminal domain of HSV-2 gG and NGF, leading to higher NGF activity and increased neurite outgrowth (21), also during infection (22). We did not investigate yet whether HSV-2 infection also modifies the protein composition of EV to overcome the repulsion of epithelial cells on neurite outgrowth.

It is plausible that HSV-1 could affect the expression of genes whose protein products modulate neurite outgrowth since this virus dramatically modifies the cell transcriptome (53). For instance, viral proteins such as virus host shutoff, ICP4 and ICP27 inhibit cellular gene expression (54–57), and this could result in different levels of proteins involved in neurite outgrowth. Whether these viral proteins and others expressed by HSV-1 play a role in the induction of neurite outgrowth upon infection of epithelial cells is currently unknown. However, our data revealed that HSV-1 gG was required and sufficient for this phenotype. HSV-1 lacking gG was less effective than its parental virus, despite similar replication in epithelial cells. Moreover, ectopic expression of HSV-1 gG inhibited the repulsion of neurite outgrowth. Therefore, despite the low sequence conservation between HSV-1 and HSV-2 gG, both proteins fulfill a similar function to enhance neurite outgrowth through paracrine mechanisms. Cleavage and secretion of HSV-2 gG allow the formation of a complex with NGF and interaction with its receptors at the neurite end (21), while HSV-1 gG is not secreted but modifies the protein composition of EV to act on distant neurites. The independent evolution of these different mechanisms underscores the relevance of neurite outgrowth for the biology of HSV-1 and HSV-2.

HSV-1 gG has been suggested to contribute to virus entry (28, 29). Moreover, several studies suggest the involvement of gG in HSV-1 neurotropism and neurovirulence in mice (30–32). The co-culture experiments in MFC together with neutralizing antibodies allowed us to mimic the *in vivo* situation of HSV-1 spread from epithelial cells into neurites, followed by retrograde transport into the neuronal cell bodies. We could therefore quantify the impact of epithelial cell infection on neurite outgrowth and determine the number of neuronal cell bodies that became infected with each virus strain. Our results showed that the increased neurite outgrowth observed when exposing SCG neurons to epithelial cells infected with parental HSV-1 resulted in higher spread to neurons. When we co-cultured HSV1-CheGL- or HSV1-CheGL-ΔgG-infected ARPE-19 cells with neurites that had already crossed the microgrooves, the number of neuronal cell bodies expressing the HSV-1 reporter mCherry was similar. This result suggests that HSV1-CheGL-ΔgG is as efficient as HSV1-CheGL in neuronal infection and axonal transport when it has equal access to neurites. Therefore, the reduced infection of neurons observed when employing epithelial cells infected with the gG-deficient virus is most likely due to its inability to attract neurites.

Previous studies already established that HSV-1 infection modifies the composition and activity of EVs. For instance, EVs from infected HSV-1 cells transfer proteins, mRNAs, and microRNAs, encoded by both host and virus, to surrounding uninfected recipient cells, playing a proviral or antiviral role depending on the specific content (58–61). We report here a novel viral manipulation of EV paracrine activity, namely to modulate their protein composition to promote neurite outgrowth and neuronal infection by HSV-1 gG. Among the enriched proteins, we focused on galectin-1, a neuroprotective and neurotrophic protein (15, 50, 62, 63). Previous work suggested that galectin-1 localizes to the surface of EVs (15) and induces neurite outgrowth upon interaction with neuropilin-1/plexinA4 receptor complex (17), expressed by mouse SCG neurons (64). Our results support that at least a fraction of galectin-1 is located on the EV surface. However, it is also possible that galectin-1 is incorporated inside EVs acting on the recipient cells following the fusion or internalization of the EVs. Such a mechanism of action has been shown for several EV proteins (65–67). The use of neutralizing antibodies confirmed that galectin-1 in EVs obtained from infected epithelial cells contributes to HSV-1 induction of neurite outgrowth. However, our results do not show that galectin-1 is the only protein required for this process. HSV-1 gG expression modified the amounts of other proteins involved in neurite outgrowth. For instance, some members of the insulin-like growth factor binding protein (IGFBP) family increase neurite outgrowth, like IGFBP2 (68), while others, like IGFBP4 and IGFBP7, decrease it (69–71). Furthermore, cathepsin D (encoded by *CTSD*) and members of the serpin and S100 superfamilies regulate neurite outgrowth (45–49) and could be involved in the phenotype reported here.

Some of the proteins enriched in VRF, or their relatives, modulate other neurological functions apart from neurite outgrowth. For instance, cystatin C (a relative of cystatin E/M encoded by *CST6*), cathepsin D, some S100 proteins, and tubulin isoforms influence neuronal activation, survival, neurodegeneration, and regulation of the cytoskeleton (45, 49, 72–77). VRF from gG-expressing cells contains less insulin-like growth factor binding protein 3 (IGFBP3), a protein that induces phosphorylation of tau, potentially playing a role in Alzheimer’s disease (78). Several reports suggest a link between HSV-1 infection and neurodegenerative disorders, including Alzheimer’s disease (for a review, see reference 27), although the involvement of HSV-1 in neurodegeneration and the potential implication of our results in this process requires further investigation. We find it particularly interesting that a protein expressed by a neurotropic virus modifies the amount of many proteins involved in neurological activities. In addition to protein components, EVs also deliver other cargos (79), such as nucleic acids (80–82), metabolites, and lipids (83) that could impact neurite outgrowth and neuronal functions that were not analyzed in this report. Further work is warranted to understand the function of the already identified proteins and other potential EV components during HSV-1 infection.

Three unsolved questions of this report are as follows: (i) what type of EVs is involved in gG activity, (ii) how does gG modify the protein composition of EVs, and (iii) is the observed effect translatable to human neurons? There are several types of EVs, including microvesicles, apoptotic bodies, and exosomes (84, 85). The size of the vesicles in the VRF (42) and their protein content, including Alix, which is involved in exosome biogenesis (86), suggest that the VRF obtained from ARPE-19 cells contains mainly exosomes. However, we cannot exclude the presence of other types of EVs in the VRF. Many cellular processes regulate EV biogenesis, cargo loading, release, and uptake by recipient cells (67). Since gG expression led to a higher amount of galectin-1 in the VRF but not in the whole-cell lysate, we hypothesize that gG modifies the sorting of proteins into the EVs through yet unknown pathways. Finally, experiments performed with human stem cell-derived neurons will determine whether EVs produced from HSV-1-infected cells increase neurite outgrowth in human neurons. These neurons could also be employed to determine the functional role of other proteins enriched in EV of gG-expressing cells. Further investigations will improve our understanding of the mechanism leading to HSV-1 gG-induced modification of the protein composition of EVs.

Artificially modified or engineered EVs are widely evaluated as therapeutic agents in cancer (87, 88), neurological (89, 90), and infectious diseases, including COVID-19 (91–93). At present, there are hundreds of registered clinical studies focusing on EVs as biomarkers, vaccine candidates, delivery vectors, or therapeutic drugs (ClinicalTrials.gov). Understanding how HSV-1 modifies the protein composition of EVs could facilitate the biogenesis and design of EVs as preventive or therapeutic drug candidates.

Overall, we demonstrate for the first time that a viral glycoprotein can modify the protein composition of EVs to increase neurite outgrowth and facilitate infection of peripheral neurons. Targeting this activity may prevent HSV-1 entry into the PNS and brain, thereby reducing pathogenesis. Moreover, the identification and functional characterization of proteins whose amount is modified by HSV-1 gG could provide novel therapeutic approaches to increase nerve regeneration and improve neuronal viability, not only after viral infection but possibly also in the context of neurodegenerative diseases.

## MATERIALS AND METHODS

### Mice

Newborn C57BL/6J mice (postnatal days 0–3) were collected from the Animal Facility of Hannover Medical School and subjected to euthanasia by trained personnel in strict accordance with the German regulations of the Society for Laboratory Animal Science, the European Health Law of the Federation of Laboratory Animal Science Association, and the German Animal Welfare Law.

### Cell lines

Human embryonic kidney HEK-293T cells (ATCC-CRL-3216) and human immortalized keratinocyte HaCaT cells (94) were cultured in Dulbecco’s modified Eagle medium (DMEM; Gibco). Human retinal pigment epithelial cell line ARPE-19 (ATCC-CRL-2302) was cultured in Dulbecco’s modified Eagle medium/Nutrient Mixture F-12 Ham (D/F-12; SIGMA). All media were supplemented with 8% fetal bovine serum (FBS; SIGMA), penicillin-streptomycin (Pen/Strep; PAN-Biotech), and 2 mM Glutamine Stable (Cytogen). *Cercopithecus aethiops* kidney epithelial Vero cells (ATCC-CCL-81) were cultured in MEM Eagle (Cytogen) with 8% FBS. Cells were grown at 37°C, with 5% CO_2_ in a humidified incubator.

### Plasmids

The coding sequence of full-length HSV-1 gG of strain 17^+^ was amplified by PCR from DNA of HSV1-CheGL-infected cells using primers FLgG-F and FLgG-R (Table 1) and cloned using In-Fusion HD Cloning Kit (Takara, Dalian, China) into pcDNA3.1Zeo(-) plasmid (Invitrogen), previously linearized with *Nhe*I and *Kpn*I, resulting in pcDNA3.1Zeo-FLgG. Galectin-1 CRISPR activation plasmids (sc-400941-ACT) and control plasmids (sc-437275) were purchased from Santa Cruz Biotechnology and diluted to 1 µg/µL with accompanying dilution water.

**TABLE 1:** Oligonucleotides employed in this study

Oligo name	Sequence (5′−3′)
HSV1-SC-fwd	GCACAAAAAGACCCCGATCCGCGTCTGTGGTGTTTTTGGCATCAAGCACGCCTAGGCTCGCGTGCCGTTGTAGGGATAACAGGGTAATCGATTT
HSV1-SC-rev	AAGAACCAAAAGGAATGGGATAATGGGAACAACGGCACGCGAGCCTAGGCGTGCTTGATGCCAAAAACGCCAGTGTTACAACCAATTAACC
HSV-1 ICP0-F	TGGACTTTATCTGGACGGGCAAT
HSV-1 ICP0-R	TCACCGTCGTCCAGGTCGT
β-actin-F	TCCTCCTGAGCGCAAGTACTCC
β-actin-R	AAGTCATAGTCCGCCTAGAAGCA
FLgG-F	ACCCAAGCTGGCTAGCATGTCGCAGGGCGCCATGC
FLgG-R	TTAAACTTAAGCTTGGTACCCTACCCGCGTTCGGACGG
MCS sense	TGAATTCTCGAGCTAGCCTGCAGGATATCACCGGTTAATTAATGCATG
MCS antisense	GATCCATGCATTAATTAACCGGTGATATCCTGCAGGCTAGCTCGAGAATTCATGCA
LGALS1-F	CATAGAAGATTCTAGAGCCACCATGGCTTGTGGT
LGALS1-R	TCGCGGCCGCGGATCCTCACTTATCGTCGTCATCCTTGT

To construct an HSV-1 gG-expressing lentivirus, a codon-optimized *US4* sequence of HSV-1 strain 17 (Gene ID: 2703404) to increase its translation in eukaryotic cells was synthesized and cloned into pUC57 plasmid (GenScript Biotech, Leiden, Netherlands, see the sequence in the supplemental material). Then, the optimized *US4* was cloned into the lentiviral-His-tagged-PSPH (Addgene plasmid #134786) using *Xba*I and *BamH*I, resulting in pLenti-FLgG-puro. A multiple cloning site sequence (generated by primers MCS sense and MCS antisense); (Table 1) was used to replace the expression cassette of lentiviral-His-tagged-PSPH *via Nsi*I and *BamH*I to get pLenti-MCS-puro, which served as lentivirus plasmid control. Human *LGALS1* ORF Clone was purchased from GenScript Biotech (Leiden, Netherlands). Then, the *LGALS1* ORF was amplified with primers LGALS1-F, and LGALS1-R (Table 1), and cloned with in-fusion cloning into lentiviral-His-tagged-PSPH previously linearized with *Xba*I and *BamH*I, resulting in pLenti-*LGALS1*-puro. The integrity of all inserts was confirmed by Sanger sequencing.

### Antibodies

Primary antibodies and dilutions were as follows: anti-HSV-1 gG Envelope Protein antibody (ab6511, Abcam) at 1:5,000; anti-beta actin monoclonal antibody (MA1-140, Invitrogen) at 1:5,000; anti-β-III-tubulin antibody (MAB5564, Sigma-Aldrich) at 1:1,000; anti-HSV-1 + HSV-2 ICP5 major capsid protein antibody (ab6508, Abcam) at 1:1,000; rabbit IgG control polyclonal antibody (30000-0-AP, Proteintech) at 1:1,000; anti-galectin-1 mouse mAb (60223-1-Ig, Proteintech) at 1:1,000; anti-galectin 1 antibody (ab25138, Abcam) at 1:1,000; anti-LGALS1 antibody (HPA000646, Sigma) at 1: 400; anti-Cas9 (7A9-3A3) mouse mAb (14,697T, CST) at 1:1,000; anti-GAPDH (14C10) rabbit mAb (2118, CST) at 1:2,000; anti-Alix polyclonal antibody (12422-1-AP, Proteintech) at 1:2,000; anti-HSP90 polyclonal antibody (13171-1-AP, Proteintech) at 1:2,000; anti-HSP70 polyclonal antibody (10995-1-AP, Proteintech) at 1:2,000; anti-FLOT1 polyclonal antibody (15571-1-AP, Proteintech) at 1:1,000; CoraLite Plus 488-conjugated galectin-1 monoclonal antibody (CL488-60223, Proteintech) at 1:100; and galectin-1 antibody (C-8) FITC (sc-166618 FITC, Santa Cruz Biotechnology) at 1:20.

Secondary antibodies and dilutions were as follows: Alexa Fluor 488 goat anti-mouse (A-11029, Invitrogen) at 1:1,000; Alexa Fluor 568 goat anti-mouse (A-11031, Invitrogen) at 1:1,000; IRDye 800CW goat anti-mouse IgG secondary antibody (926-32210, LI-COR) at 1:10,000; IRDye 680RD goat anti-rabbit IgG secondary antibody (926-68071, LI-COR) at 1:10,000.

### Viruses

The previously characterized reporter virus HSV-1(17^+^)Lox-_pHCMV_mCheGLuc (37), termed HSV1-CheGL in this manuscript, was used to generate HSV-1(17^+^)Lox-_pHCMV_mCheGLuc-ΔgG (termed HSV1-CheGL-ΔgG) by *en-passant* mutagenesis (35, 36), using primers HSV1-SC-fwd and HSV1-SC-rev (Table 1). Both viruses express mCherry and *Gaussia* luciferase, separated by a P2A site, driven by the human cytomegalovirus major immediate early promoter. We deleted the ATG of the *US4* gene encoding for gG and added a frameshift and a stop codon to reduce the risk that the expression of gG would be rescued by random mutagenesis (Fig. S2A). The insertion of mutations and the genome integrity of the recombinant virus were confirmed by next-generation sequencing of the viral genome with a MiSeq device (Illumina, Inc.) as previously described (22) (sequence submitted to GenBank under accession number OP950204). Lack of gG protein expression was determined by western blotting.

### DNA transfection

For transient transfections, 6 × 10^5^ ARPE-19 or 1.2 × 10^6^ HEK-293T cells per well were seeded in 6-well plates. On the following day, cells were transfected with 1 or 2 µg (for ARPE-19 or HEK-293T cells, respectively) plasmid DNA per well using *Trans*IT-X2 (Mirus Bio, Madison, WI, USA). Medium change was performed 6 hours later. Transfections to generate lentiviruses are explained in “Production of lentiviruses and generation of stably transduced cells.”

### Production of lentiviruses and generation of stably transduced cells

For lentivirus production, 5 µg pLenti-FLgG-puro or pLenti-LGALS1-puro, 3.5 µg p8.91, and 1.5 µg pVSV-G (95) were co-transfected with *Trans*IT-X2 into a monolayer of HEK-293T cells in P100 cell culture dish (seeded at about 5 × 10^6^ cells one day ahead). Six hours later, the medium was replaced with 10 mL fresh growth medium plus 25 mM HEPES. Forty-eight hours later, the conditioned medium containing lentivirus was collected and filtered through a 0.22-µm filter. The resulting lentivirus supernatant was used directly or stored at −80°C. For lentiviral transduction of ARPE-19 cells, confluent cells in a P150 dish were split into 2 P150 dishes and incubated with growth medium plus 1 mL fresh lentivirus supernatant. After 24 hours, 10 µg/mL puromycin was added to select for transduced cells. After three passages under antibiotic selection, the antibiotic-resistant cells were used for experiments.

### Immunofluorescence microscopy

The cells in MFC were fixed with 4% paraformaldehyde in PBS (wt/vol) at RT for 30 min and then permeabilized using 0.2% Triton X-100 in PBS (vol/vol) for 30 min, followed by incubation with blocking buffer (PBS containing 3% [wt/vol] bovine serum albumin) (IF grade, IgG free; Gibco) for 1 hour. The cells in MFC were then incubated with primary antibodies diluted in a blocking buffer at 4°C overnight. On the following day, the cells were washed and incubated with secondary antibodies and DAPI (1:500) in a blocking solution at RT for 2 hours. After incubation, the cells in MFC were washed again and mounted with 50% glycerol in PBS. Stable ARPE-19 cells were seeded on coverslips, then fixed and stained as indicated for MFC, except that the incubation time of a secondary antibody was reduced to 1 hour. Stained coverslips were sealed on the slide with ProLong Gold Antifade Mountant (Thermo Fisher Scientific) and then incubated at 4°C overnight. Images were acquired using a Zeiss Observer Z1 inverted microscope for MFC and coverslips.

SCG explants were fixed using 4% paraformaldehyde in PBS (wt/vol) at RT for 1 hour, permeabilized using 0.5% Triton X-100 in PBS (vol/vol) for 1 hour, and then blocked with PBS plus 3% bovine serum albumin (IF grade, IgG free; Gibco) for 1 hour. Primary antibodies in a blocking buffer were incubated overnight followed by a 1-hour incubation with secondary antibodies and DAPI counterstaining in a blocking buffer, all at RT. After mounting with 50% glycerol in PBS, the SCG explants were imaged using an FV1000 confocal laser scanning microscope (Olympus) from top to bottom taking images every 10 µm. Intensity projection over the Z-axis was built for further analysis with Fluoview viewer V4.2b.

### Western blotting

Cells and VRF were lysed in RIPA buffer with protease inhibitor cocktail (Thermo Fisher Scientific) for 15 min on ice. The cell and VRF lysates were collected after centrifugation at 17,000 × *g* for 10 min and mixed with the SDS loading buffer, while TCM and VFF were directly mixed with the loading buffer. Samples were heat denatured at 98°C for 5 min, centrifuged for 1 min at 17,000 × *g*, and loaded into SDS-PAGE gels. The separated proteins were transferred onto nitrocellulose membranes. The membranes were incubated in blocking buffer (PBS containing 0.1% Tween 20 [PBS-T] and 5% skimmed milk) for 2 hours followed by overnight incubation at 4°C with primary antibody diluted in blocking buffer. After three washes in PBS-T, the membranes were incubated with fluorescently conjugated secondary antibody diluted in blocking buffer for 1 hour at room temperature (RT). Detection was performed with ChemiDoc MP Imaging System (Bio-Rad). Original western blots are shown in Fig. S10.

### Neurite outgrowth assay with SCG explants

Mouse SCGs were dissected from newborn mice as previously described (96, 97) and cultured in a 3D collagen matrix as described before (21). To test neurite outgrowth, DMEM or D/F12 (neuron culture medium employed as normal control, neuron medium), TCM, VFF, and VRF were supplemented with 10% FBS, 1% Pen/Strep, and 25 pM mouse NGF 2.5S (G514A, Promega) and added to SCG explants. The generation of TCM, VFF, and VRF is explained in “Fractionation of conditioned medium,” below. In the neutralization test, VRF and antibodies (1 µg/mL) were incubated for 30 min before use. After 20–24 hours of incubation with TCM, VFF, or VRF, the SCG explants were fixed using 4% paraformaldehyde in PBS (wt/vol) at RT for 1 hour, and immunofluorescence microscopy was performed (see “Immunofluorescence microscopy”).

### Production of virus stocks

The virus stocks were produced from the supernatant and lysate of infected Vero cells. Briefly, twenty 15 cm dishes with Vero cells were infected at an MOI of 0.01 and incubated for at least 72 h at 37°C, until a full cytopathic effect was visible. Then, the cells were scraped off and pelleted into 50 mL Falcon tubes. The supernatant was kept on ice, while the cell pellets were disrupted by three freeze-thaw cycles. The cell debris was then pelleted in a Heraeus tabletop centrifuge (860 × *g*, 5 min, 4°C), and the supernatant was then combined with the supernatant from the first round of centrifugation. The virus was then pelleted using a Beckman L8-70 ultracentrifuge (type 19 rotor, 12,000 rpm, 4°C, 90 min), and the pellet was resuspended in 1 mL of cell culture medium.

### HSV-1 replication kinetics

To determine replication kinetics in ARPE-19 by quantification of genome copy number, 3 × 10^5^ ARPE-19 cells per well were seeded in 12-well plates. After overnight culture, cells were incubated with virus inoculum (HSV1-CheGL or HSV1-CheGL-ΔgG virus stocks diluted in 2% FBS medium, MOI of 0.01) at 37°C for 1 hour. The cells were then washed with PBS and cultured with 2% FBS medium. At the indicated time points, cells were harvested for DNA extraction, and quantitative real-time PCR (qPCR) was performed. To determine HSV-1 replication kinetics in Vero cells, confluent cell monolayers in 12-well plates were infected with virus inoculum at an MOI of 0.01. Then, the cells were overlaid with 1 mL of fresh DMEM containing 2% FBS. At the time points indicated in the Results section, supernatant and cells (subjected to three freeze-thaw cycles) from two wells per virus strain were harvested and titrated on Vero cells in duplicate. To do so, confluent Vero cells were inoculated with 10-fold serial dilutions of the collected supernatant in MEM Eagle containing 2% FBS, incubated for 1 hour at 37°C, washed with PBS, and overlayed with MEM Eagle containing 2.5% FBS and 0.3% (wt/vol) of carboxy-methyl-cellulose. Plaques were counted using a light microscope at 2 dpi.

### HSV-1 virion purification

Gradient purification of HSV-1 virions was performed as previously described (41). Supernatant from infected HaCaT cells was centrifuged in a Beckman L8-70 ultracentrifuge (type 19 rotor, 12,000 rpm, 4°C, 90 min) to obtain cell-free virions. The pellet was resuspended in 1 mL of PBS, then further purified by ultracentrifugation (SW40 rotor, 14,000 rpm, 4°C, 60 min) through a 10% nycodenz cushion in PBS. The pellet was resuspended in 1 mL of PBS again and followed by sedimentation in a linear nycodenz gradient (10%–40%) by ultracentrifugation (SW40 rotor, 20,000 rpm, 4°C, 120 min). The generated visible bands were collected without disturbing the gradient and the presence or absence of viral and EV proteins was determined by western blotting. The samples were UV inactivated and employed in neurite outgrowth assay.

### DNA extraction and qPCR

Total DNA was extracted from eukaryotic cells employing the QIAamp DNA Blood Mini Kit (QIAGEN), following the manufacturer’s instructions. Luna Universal qPCR Master Mix (NEB) was used to prepare the reaction mixture. HSV-1 *ICP0* served as the viral genome target, amplified with primers HSV-1 ICP0-F and HSV-1 ICP0-R (Table 1), while human β*-actin* served as the host genome target for normalization, amplified with primers β-actin-F and β-actin-R (Table 1). The PCR products were cloned into pGEM-T Easy Plasmid (Promega) and verified by Sanger sequencing. Serial dilutions of plasmid DNA were used as qPCR templates to generate standard curves. The amplification and detection were performed using a qTOWER³ Real-time Thermal Cycler (Analytik Jena). The copy number of the target gene in the samples under study was calculated from the standard curves.

### *Gaussia* luciferase activity assay

To quantify *Gaussia* luciferase (GLuc) activity in infected cell cultures, we used a microplate luminometer (Orion II; Berthold) with an injector system, as described before (22). Briefly, 50 µL cell culture supernatant was placed into a 96-well opaque white plate (Nunc) and was mixed with 50 µL of PBS containing 1 µg/mL native coelenterazine (Sigma) prior to quantification.

### Neurite outgrowth and infection assay in MFC

The dissociation and culture of primary SCG neurons, as well as the co-culture of neurons and ARPE-19 cells were adapted from procedures described before (22). Briefly, the SCGs were collected and dissociated by sequential digestions with papain (30 min at 37°C) and a dispase-collagenase mixture (30 min at 37°C), followed by disruption with 1 mL pipette tips. The cells were washed and resuspended in 5 µL neuron medium per MFC (3 SCG/MFC). Then, the neuron suspension was added into the somal compartment (SC) of microfluidic chambers (MFC) that contain 450 µm long microgrooves (RD450; Xona Microfluidics, USA).

To determine the relevance of infection in neurite outgrowth, 1.5 × 10^6^ HEK-293T or 6 × 10^5^ ARPE-19 cells per well were seeded in six-well plates. The next day, the cells were washed and overlaid by 1 mL viral inoculum (HSV1-CheGL or HSV1-CheGL-ΔgG virus stocks diluted in 2% FBS medium, MOI of 1) or 2% FBS medium as mock infection control and incubated for 1 hour at 37°C. Afterward, the cells were washed with PBS and further incubated in normal culture medium for 16 hours. Mock- or HSV-1-infected ARPE-19 cells were scraped off six-well plates, pelleted, and resuspended in 120 µL neuron medium. The neuron medium employed in these experiments to seed SCG and ARPE-19 cells contained HSV-1 neutralizing human immunoglobulins (1:100; CSL Behring GmbH, Marburg, Germany) to prevent direct infection of neuronal cell bodies due to diffusion of cell-free virus, allowing only cell-to-cell spread (41). 5 µL of ARPE-19 cell suspension (5 × 10^4^ cells) was added to the neurite compartment (NC) immediately or 24 h post-seeding of the dissociated SCG neurons, according to the specific experiment, as explained in the Results section and figure legends. An additional 15 µL of cell suspension was added to each reservoir well at the NC so that the factors released by epithelial cells could diffuse to the SC. Following 20–24 hours of incubation, the cells were fixed and labeled with antibodies or stained with DAPI (see “Immunofluorescence microscopy”).

### Quantification of neurite outgrowth and neuronal infection

FIJI software (98) was used to quantify neurite outgrowth. In short, the projection images of SCG explants were loaded into the FIJI software and split into single-channel images, and the channel corresponding to β-III-tubulin staining was used to measure the fluorescence intensity of neurites, while the DAPI channel was used to measure the fluorescence intensity of SCG nuclei. After background subtraction, the intensity ratio of β-III-tubulin staining to DAPI staining, indicative of the amount of cytoskeleton protein (and thereby, neurites) per nuclei intensity was calculated and used for further statistical analysis.

To quantify neurite outgrowth and neuronal infection of dissociated neurons, images were split into single-channel images. mCherry and β-III-tubulin channels were used to quantify neuronal infection at the SC side of MFC and neurite outgrowth at the NC side, respectively. To quantify neurite outgrowth, plug-in NeuronJ (99) was used. The neurite paths were distinguished, then the total number and length of marked neurite paths of each image were measured.

### Fractionation of conditioned medium

Transiently transfected cells were incubated in growth medium for 24 hours after transfection, then the cells were washed and the medium was changed to 1 mL pure medium (DMEM for HEK-293T, D/F12 for ARPE-19) without any additives. After 24 hours, the conditioned medium was collected. In infection conditions, the infected cells were washed immediately after 1-hour virus incubation, and 1 mL pure medium (DMEM for HEK-293T, D/F12 for ARPE-19) without any additives was added. After 16 hours, the conditioned medium was collected and inactivated by ultraviolet light. Virus inactivation was determined by inoculating naïve Vero cells and assessing for lack of cytopathic effect. Stably transduced ARPE-19 cells were seeded in six-well plates at 6 × 10^5^ cells per well and cultured overnight. The next day, the cells were washed and 1 mL of pure D/F12 medium without any additives was added. After 24 hours, the conditioned medium was collected.

Irrespective of the cellular origin or whether it was obtained from transfected, transduced, or infected cells, the conditioned media were further processed at 4°C, following a previously described protocol (42) with adaptations (Fig. 4A). First, the conditioned media were centrifuged at 2,000 *× g* for 5 min to remove large cell debris. Then 10 min of centrifugation at 17,000 × *g* was applied to further remove small cell fragments and large EV from the conditioned medium. The resulting supernatant was named total conditioned medium (TCM). To prepare the vesicle-free fraction (VFF) and vesicle-enriched fraction (VRF), the TCM harvested from ARPE-19 cells was dropwise filtered using 0.22 µm filter (Merck Millipore Ltd.), and further ultracentrifuged at 120,000 × *g* for 2 hours using TLA 120.2 rotor with Optima MAX-XP ultracentrifuge (Beckman Coulter Life Sciences). The supernatant was termed VFF, which mainly contains secreted soluble factors. The pellet was resuspended with pure D/F12 and termed VRF.

### Nanoparticle analysis

About 200 µL of VFF or VRF was injected with a 1 mL sterile syringe into the sample chamber of a NanoSight LM10 instrument (Malvern Panalytical Ltd, UK). The vesicles in the laser beam underwent Brownian motion and 60 second video of the movements was recorded. Then the NanoSight NTA software (v3.4.4) analyzed the video and determined the concentration and size distribution of the vesicles in the samples.

### Flow cytometry analysis of EVs

To detect galectin-1 on EV surfaces, the FACS assay of EVs was performed as described before (100). VRFs were prepared from supernatant of vector- and galectin-1-transduced ARPE-19 cells, resuspended in 100 µL of PBS and incubated with conjugated galectin-1 antibody (10 µg/mL) or PBS with 2% BSA, rotating for 2 hours on ice. Afterward, the EVs were diluted in 1 mL PBS and ultracentrifuged at 120,000 × *g* for 1 hour to remove unbound antibodies. EVs were resuspended in 100 µL PBS and analyzed by flow cytometry with a CytoFLEX S (Beckman Coulter). Data analysis was performed with FlowJo Software (BD Life Sciences).

### Transmission electron microscopy

Negative staining of the VRF was performed as described before (101, 102). Briefly, 5 µL of VFF or VRF was adsorbed for 20 min at RT onto enhanced hydrophilicity-400 mesh copper grids (Electron Microscopy Sciences, PA, USA). The grids were then washed with PBS and ddH_2_O, contrasted with 2% uranyl acetate at pH 4.4, air-dried, and then analyzed by transmission electron microscopy (Morgani 268 at 80 kV; FEI, Eindhoven, The Netherlands).

### Mass spectrometry and data analysis

VRF samples were diluted in 2 × lysis buffer (2% sodium deoxycholate, 100 mM Tris-HCl pH 8, 2 mM EDTA, 20 mM dithiothreitol, 80 mM chloroactamide; Sigma), heated for 10 min at 95°C and cooled down. To each sample, 1 µg sequence grade trypsin (Promega) and 1 µg LysC (Wako) were added and incubated overnight at 37°C. After acidifying the samples with formic acid (1% final concentration), peptides were extracted and cleaned up using the stage tips protocol (103). Peptides were eluted from stage tips (80% acetonitrile and 0.1% formic acid), dried using the speed vac system, resolved in 3% acetonitrile/0.1% formic acid, and injected into the LC-MS/MS system (Thermo Scientific).

Raw data were processed using the MaxQuant software package v1.6.3.4 (104). The internal Andromeda search engine was used to search MS2 spectra against a decoy human UniProt database (HUMAN.2019-07) and human herpes simplex Uniprot database (9294) containing forward and reverse sequences. The false discovery rate (FDR) was set to 1% for peptide and protein identifications. Unique and razor peptides were considered for quantification. Five replicates were assigned to each experimental group and data were filtered for a minimum value of three in at least one group, and for proteins with a minimum of three MS/MS counts. Differential protein abundance was calculated using Student’s *t*-test. Top-regulated proteins were defined with a cutoff of 15% FDR. The mass spectrometry proteomics data have been deposited to the ProteomeXchange Consortium via the PRIDE (105) partner repository with the data set identifier PXD039569.

### Statistical analysis

All statistical analyses were performed using GraphPad Prism 9 (GraphPad Software, San Diego, CA, USA). Outliers were identified by ROUT (Q = 1%). Normality and lognormality tests were performed to check data distribution. Statistical significance was calculated using an unpaired *t*-test (normal distribution) or Mann-Whitney test (nonnormal distribution) for two groups, while one-way ANOVA (normal distribution) or Kruskal-Wallis tests (nonnormal distribution), with Dunn’s multiple comparisons test, were applied for data sets with three or more groups, as indicated in relevant figure legends. *P* values less than 0.05 were considered statistically significant. *P* > 0.05, ns (not significant); *P* < 0.05 *; *P* < 0.01 **; *P* < 0.001 ***; *P* < 0.0001 ****. The statistical analysis performed to analyze the mass spectrometry results is explained in “Mass spectrometry and data analysis.”

## Data Availability

The data supporting the findings of this study are available within the article and its supplemental material. Mass spectrometry raw data and protein output tables are available via ProteomeXchange with the identifier PXD039569.

## SUPPLEMENTAL MATERIAL

Table S1 mbio.03308-23-s0001.xlsx Normalized quantitative protein data from our mass spectrometry-based analyses.

Supplemental Figures mbio.03308-23-s0002.pdf Figures S1 to S10.

Supplemental Figure Legends mbio.03308-23-s0003.docx Legends for the supplemental figures.

Supplemental Table Legend mbio.03308-23-s0004.docx Legend to Table S1.

ASM does not own the copyrights to Supplemental Material that may be linked to, or accessed through, an article. The authors have granted ASM a non-exclusive, world-wide license to publish the Supplemental Material files. Please contact the corresponding author directly for reuse.
