# Supplementary material for: Herpes simplex virus type 1 modifies the protein composition of extracellular vesicles to promote neurite outgrowth and neuroinfection
Source: mBio. 2024 Jan 26;15(2):e03308-23. doi: 10.1128/mbio.03308-23 (PMC10865794; doi:10.1128/mbio.03308-23)

S1 Figure

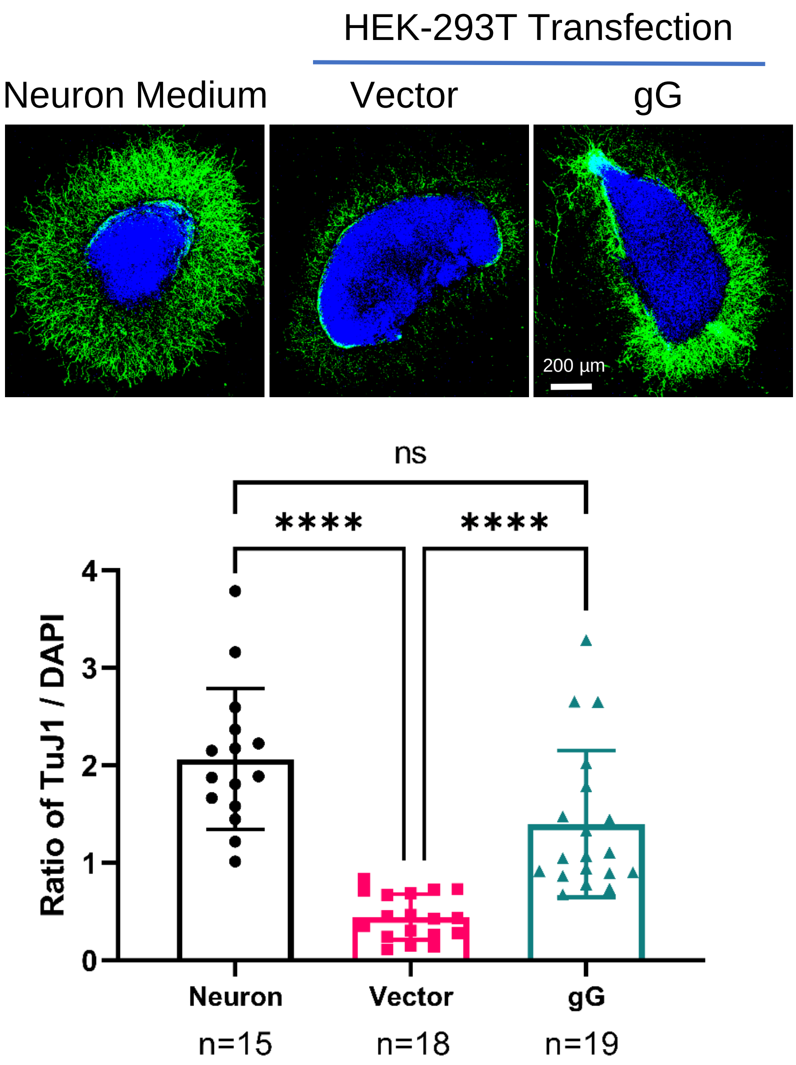

HEK-293T Transfection
Neuron Medium
Vector
gG
200 µm
ns
****
****
Ratio of TuJ1 / DAPI
0
1
2
3
4
Neuron
Vector
gG
n=15
n=18
n=19

S2 Figure

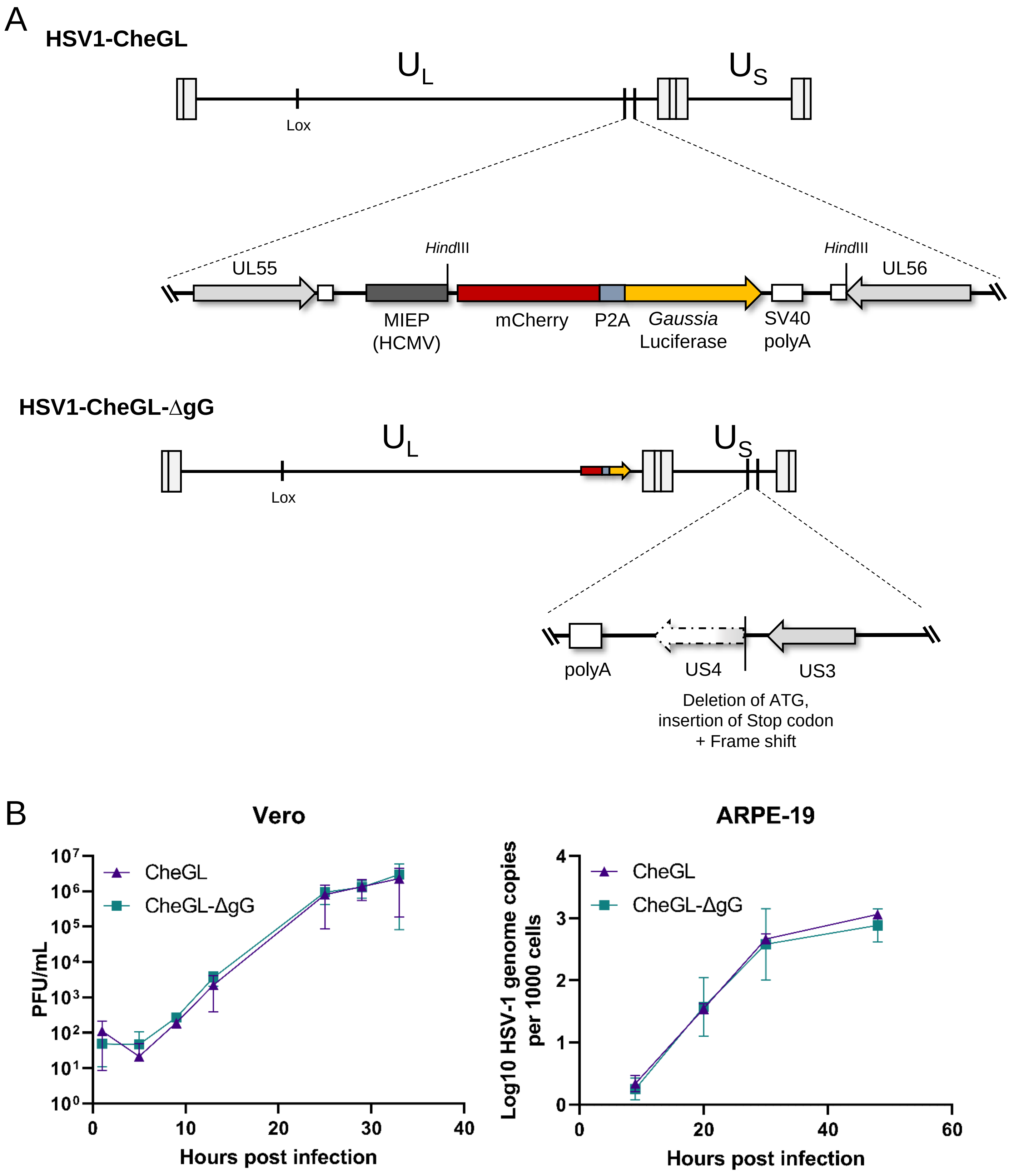
A
HSV1-CheGL
UL
US
Lox
HindIII
HindIII
UL55
UL56
MIEP (HCMV)
mCherry
P2A
Gaussia Luciferase
SV40 polyA
HSV1-CheGL-ΔgG
UL
US
Lox
polyA
US4
US3
Deletion of ATG, insertion of Stop codon + Frame shift
B
Vero
PFU/mL
CheGL
CheGL-ΔgG
Hours post infection
ARPE-19
Log10 HSV-1 genome copies per 1000 cells
CheGL
CheGL-ΔgG
Hours post infection

S3 Figure

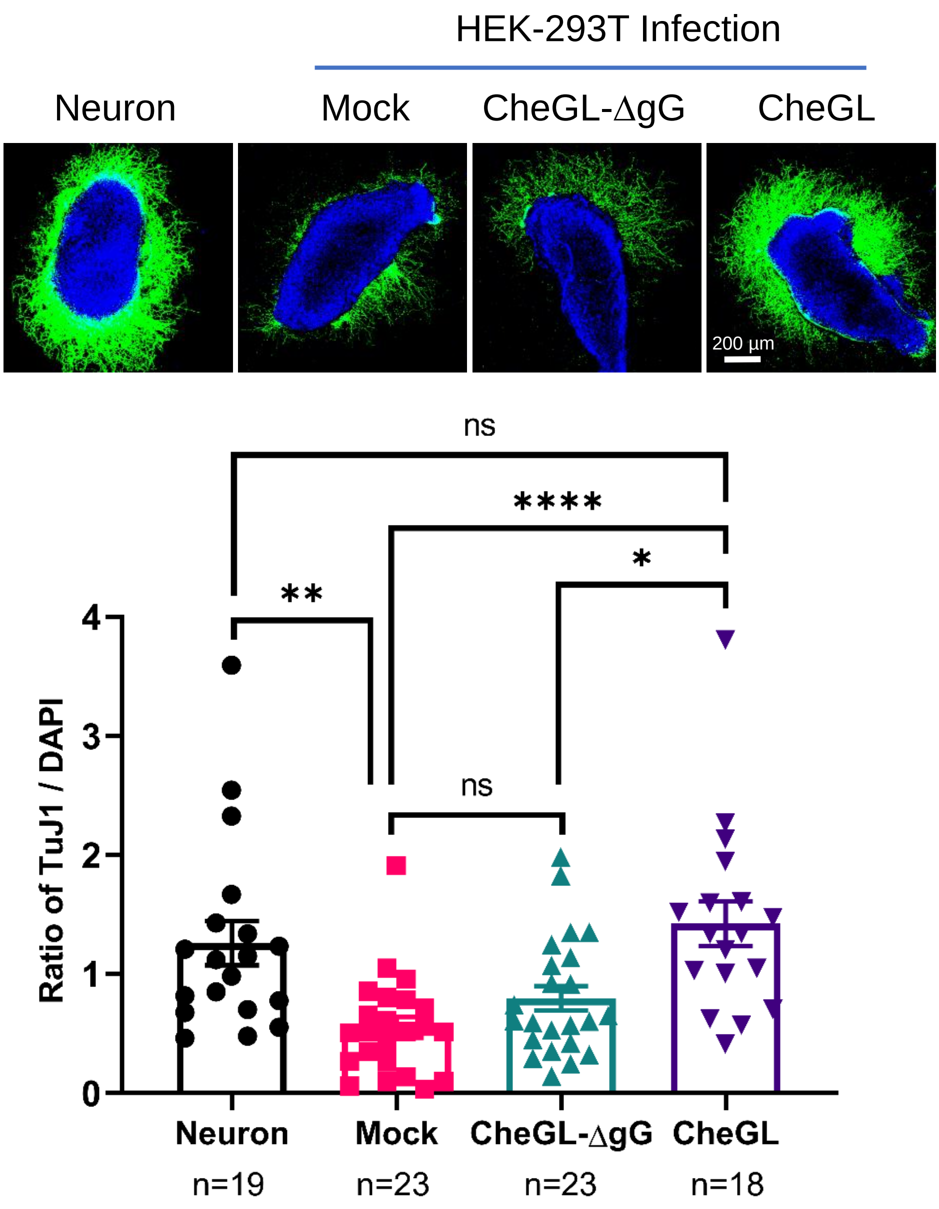
HEK-293T Infection
Neuron
Mock
CheGL-ΔgG
CheGL
200 µm
ns
****
*
**
ns
Ratio of TuJ1 / DAPI
4
3
2
1
0
Neuron
Mock
CheGL-ΔgG
CheGL
n=19
n=23
n=23
n=18

S4 Figure

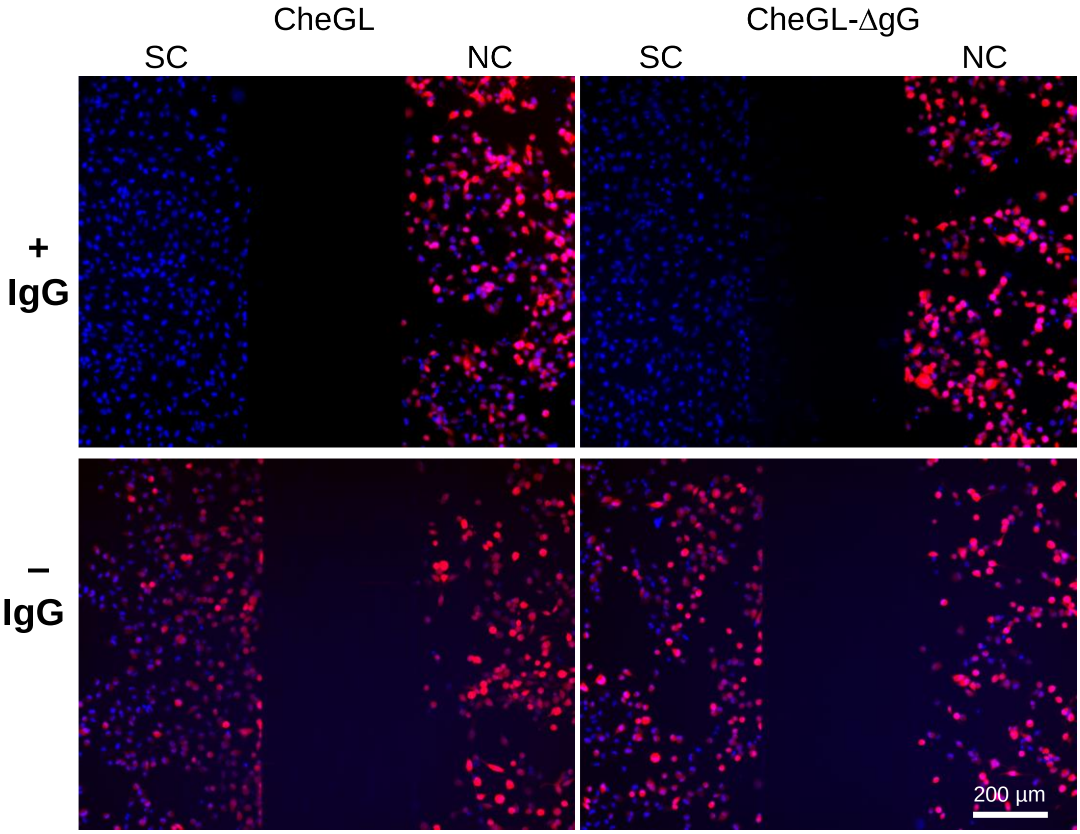

S5 Figure

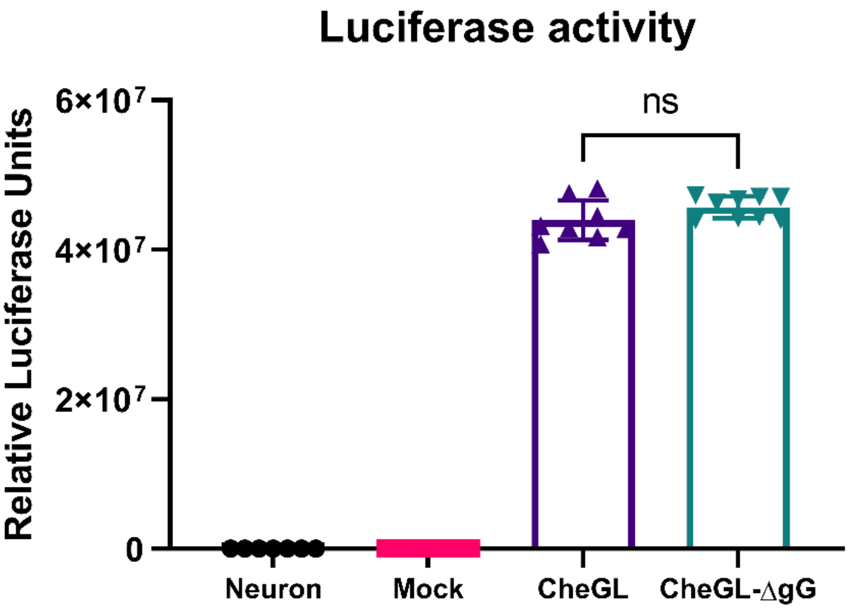
Luciferase activity
ns
Relative Luciferase Units
6×10⁷
4×10⁷
2×10⁷
0
Neuron
Mock
CheGL
CheGL-ΔgG

S6 Figure

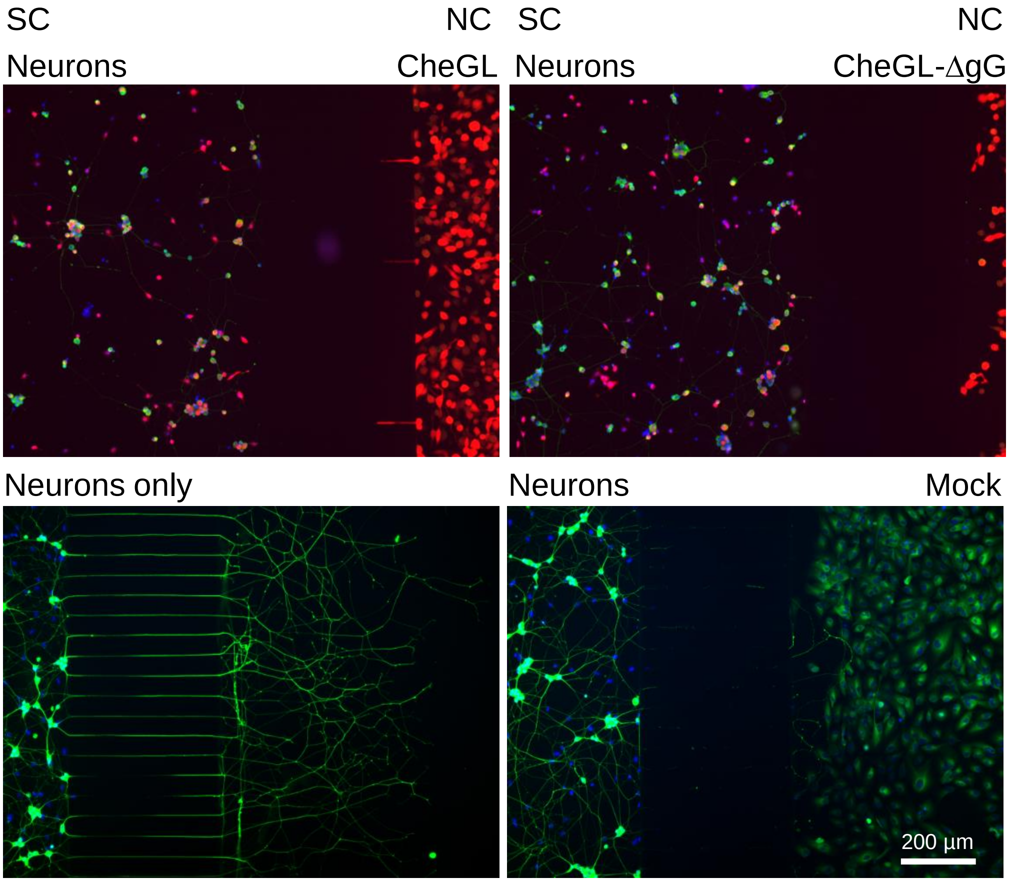
SC
NC
Neurons
CheGL
SC
NC
Neurons
CheGL-ΔgG
Neurons only
Neurons
Mock
200 µm

S7 Figure

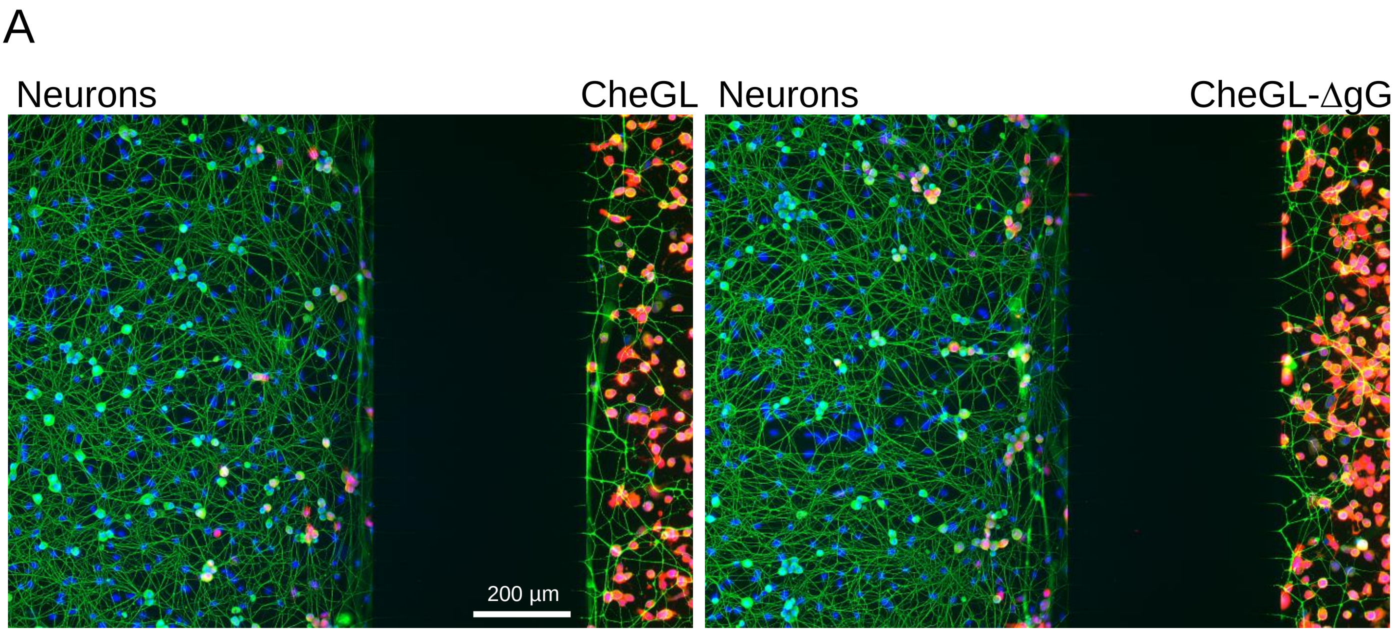
A
Neurons
CheGL
Neurons
CheGL-ΔgG
200 µm

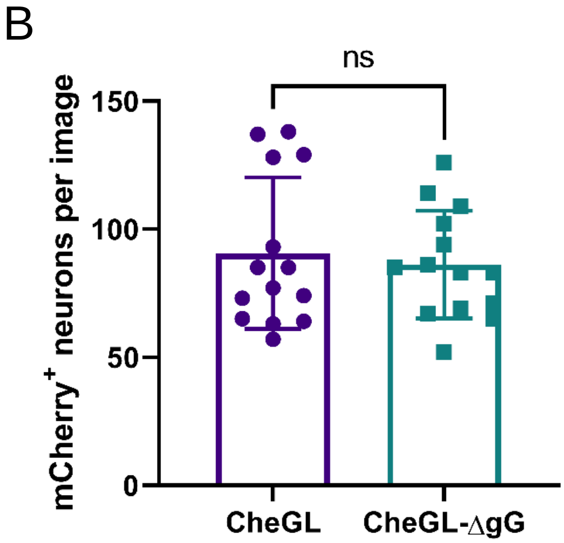
B
ns
mCherry+ neurons per image
150
100
50
0
CheGL
CheGL-ΔgG

S8 Figure

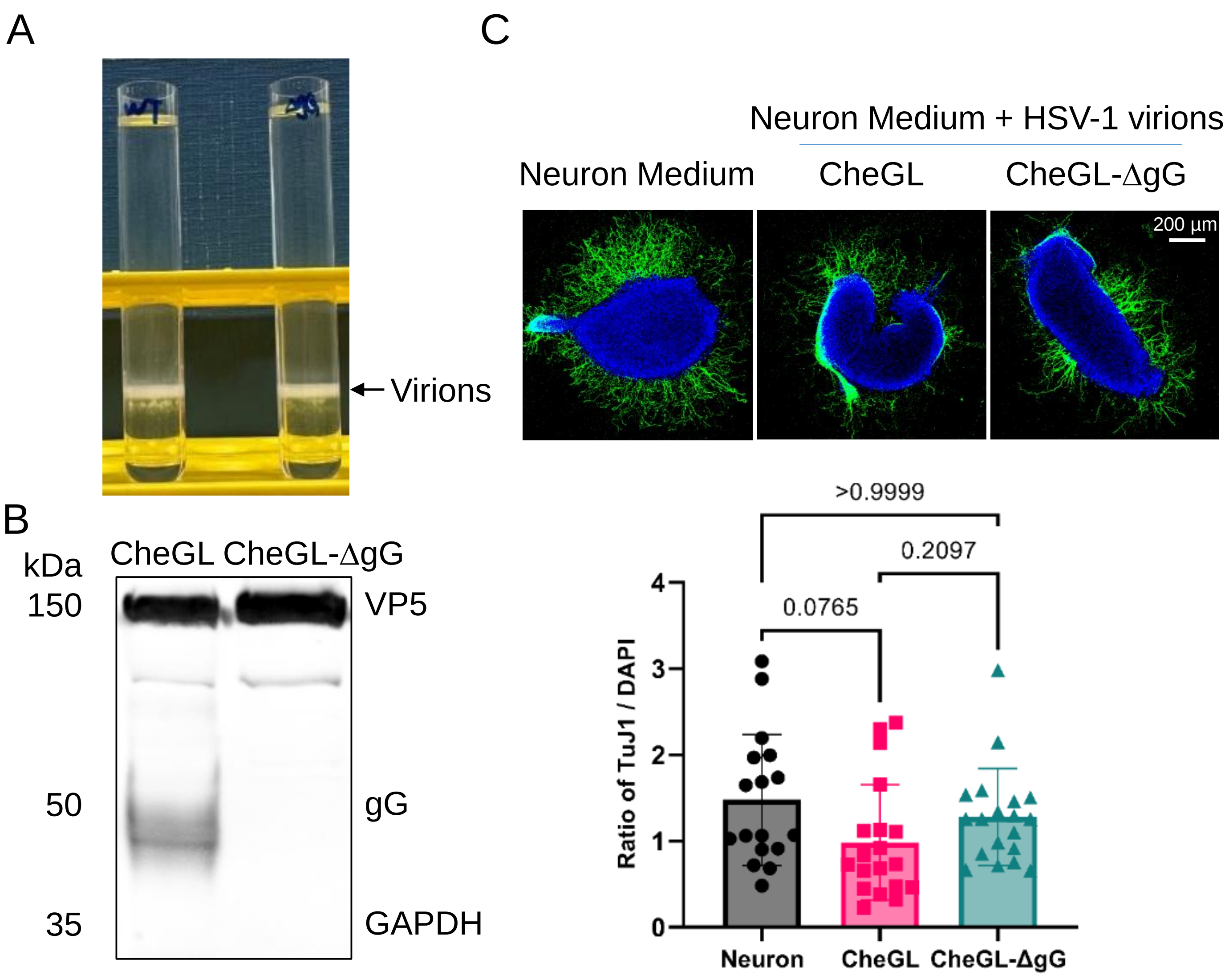
A
Virions
B
kDa
CheGL CheGL-ΔgG
150
50
35
VP5
gG
GAPDH
C
Neuron Medium + HSV-1 virions
Neuron Medium
CheGL
CheGL-ΔgG
200 µm
>0.9999
0.2097
0.0765
Ratio of TuJ1 / DAPI
0
1
2
3
4
Neuron
CheGL
CheGL-ΔgG

S9 Figure

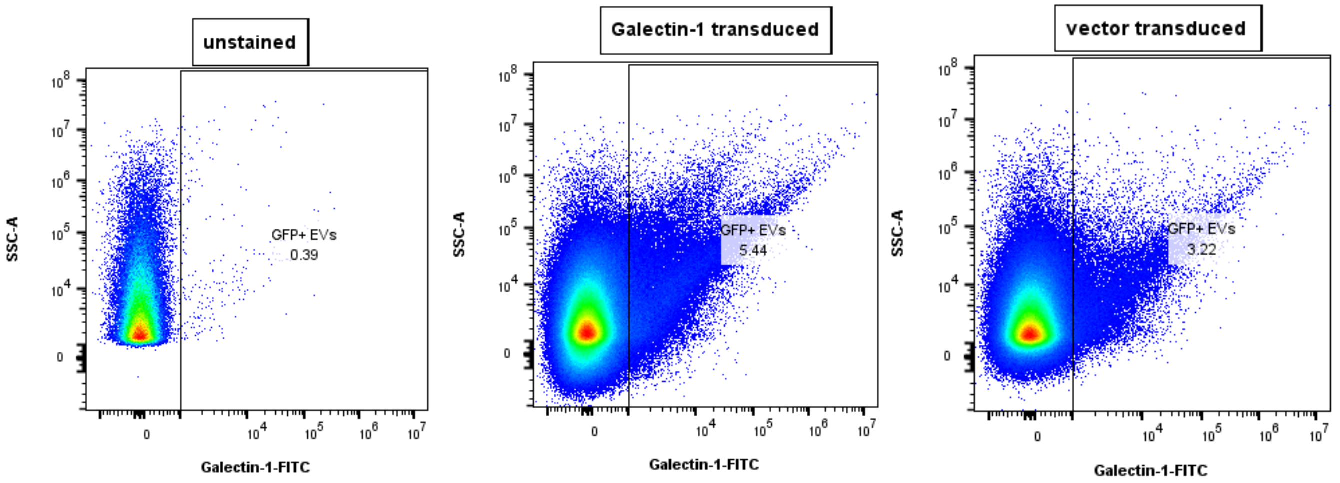

unstained
Galectin-1 transduced
vector transduced
SSC-A
Galectin-1-FITC
GFP+ EVs
0.39
5.44
3.22

S10 Figure

Fig. 4D

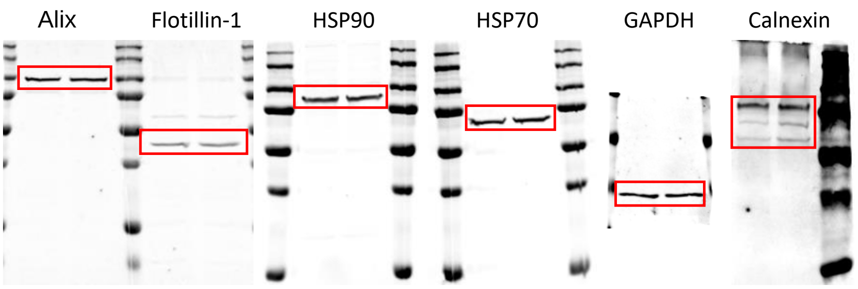

Fig. 4E

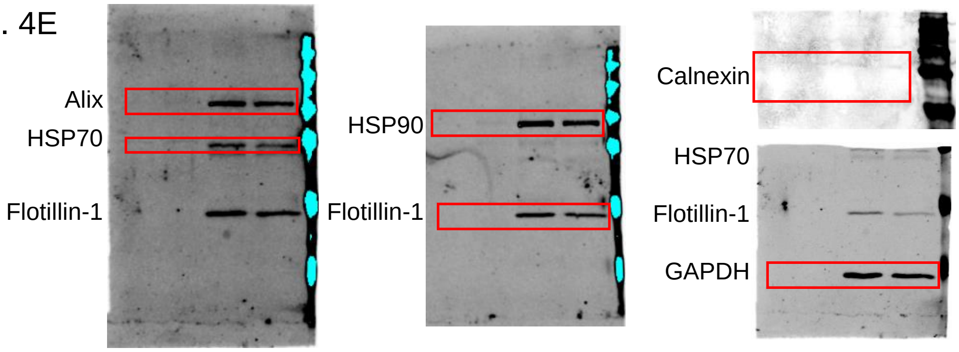

Fig. 6C

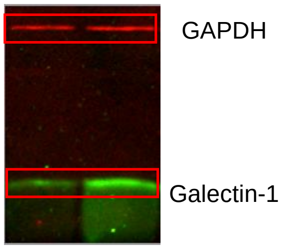

Fig. 6D

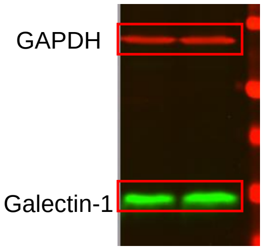

Fig. 7A

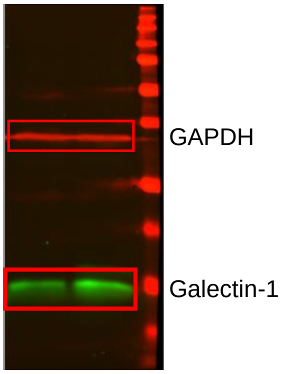

Fig. 7B

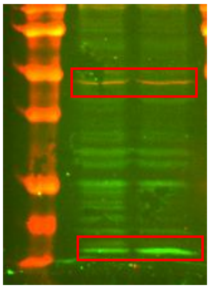

Fig. 7C

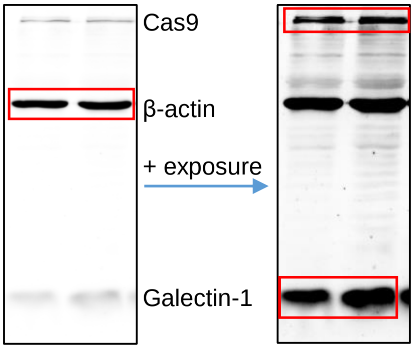

Fig. 7D

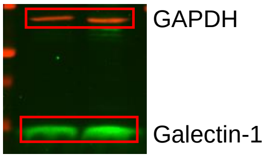

Supplement: Supplemental Figures — Figures S1 to S10. [file mbio.03308-23-s0002.pdf]
