# Supplementary material for: Herpes simplex virus type 1 modifies the protein composition of extracellular vesicles to promote neurite outgrowth and neuroinfection
Source: mBio. 2024 Jan 26;15(2):e03308-23. doi: 10.1128/mbio.03308-23 (PMC10865794; doi:10.1128/mbio.03308-23)
Supplement: Supplemental Figure Legends — Legends for the supplemental figures. [file mbio.03308-23-s0003.docx]

**Supplemental Figure Legends:**

**Figure S1. HSV-1 gG inhibits the repelling effect of HEK-293T cells on neurite outgrowth.** Top panels show representative confocal microscopy images of SCG incubated with neuron medium or conditioned medium from transiently transfected HEK-293T cells, supplemented with serum, NGF and antibiotics. After 20-24 hours, SCG were fixed and labelled with anti-β-III-tubulin antibody (TuJ1, green) and stained with DAPI (blue). Scale bar: 200 μm. The graph below shows the quantification of neurite outgrowth in each experimental group, presented as the ratio of neurite fluorescence intensity (TuJ1) to DAPI signal intensity for each SCG. Quantification was performed with FIJI software (see Materials and Methods). Each symbol corresponds to one SCG. N indicates the number of SCG in each experimental condition. Data are presented as mean ± standard deviation of the mean. Ns, not significant; **** *p* < 0.0001 (Kruskal-Wallis test with Dunn's multiple comparisons test).

**Figure S2. Generation and characterization of HSV1-CheGL-ΔgG.** (**A**) Schematic representation of HSV1-CheGL (top) and HSV1-CheGL-ΔgG (bottom), expressing both mCherry and *Gaussia* luciferase from the major immediate early promoter (MIEP) of human cytomegalovirus (HCMV). The unique long (UL) and unique short (US) regions, the internal and terminal repeats, and the inserted Lox site are shown. The enlarged region below HSV1-CheGL shows the mCherry-P2A-GLuc reporter cassette between ORFs *UL55* and *UL56*. The enlarged region below HSV1-CheGL-ΔgG depicts the mutations introduced in *US4*, the gene encoding gG, to abrogate its expression. (**B**) Graph showing replication kinetics of HSV1-CheGL and HSV1-CheGL-ΔgG in Vero (left) and ARPE-19 (right) cells. Left graph: Triplicate wells of Vero cells were infected with both HSV-1 strains and supernatants were collected at the indicated times. The collected samples were then employed to perform plaque assays on naïve Vero cells. Right graph: Three wells of ARPE-19 cells were infected with both HSV-1 strains and lysed at the indicated times. Total DNA was extracted and used for qPCR analysis. HSV-1 genome copy number was quantified by amplifying ICP0, while the host genome was quantified by amplifying β-actin. Genome copy numbers were calculated by generating standard curves and presented as ratio of ICP0 to β-actin. Data are presented as mean ± standard deviation of the mean. Abbreviations: SV40, simian virus 40; PFU, plaque forming units.

**Figure S3. HSV-1 gG inhibits the repelling effect of HEK-293T cells on neurite outgrowth.** Top panels show representative immunofluorescence confocal images of SCG incubated with neuronal medium or conditioned medium from mock- or HSV-1-infected HEK-293T cells. The media were treated with ultraviolet light (UV) to inactivate HSV-1. After 20-24 hours, SCG were fixed and labelled with anti-β-III-tubulin antibody (TuJ1, green) and stained with DAPI (blue). Scale bar: 200 μm. The graph below shows the quantification of neurite outgrowth in each experimental group, presented as the ratio of neurite fluorescence intensity (TuJ1) to DAPI signal intensity for each SCG. Quantification was performed with FIJI software (see Materials and Methods). Each symbol corresponds to one SCG. N indicates the number of SCG in each experimental condition. Data are presented as mean ± standard error of the mean. N=ganglion number. Ns, not significant; *p* < 0.05 *; *p* < 0.01 **; *p* < 0.0001 **** (Kruskal-Wallis tests with Dunn's multiple comparisons test).

**Figure S4. HSV-1 neutralizing antibodies inhibit cell-free virus infection of cells seeded in the NC.** Uninfected and infected ARPE-19 cells were seeded on the somal compartment (SC) and neurite compartment (NC) of MFC, respectively, in the presence or absence of HSV-1 neutralizing human immunoglobulins (IgG, top panels and bottom panels, respectively). Cells were fixed at 24 hours post-seeding. Nuclei were stained with DAPI (blue), and infected cells were detected by mCherry expression (red). Scale bar: 200 µm.

**Figure S5. ARPE-19 cells infected with HSV1-CheGL and HSV1-CheGL-ΔgG secrete similar luciferase levels.** Graph showing relative luciferase levels quantified in the supernatant of infected ARPE-19 cells collected at the endpoint of the experiment, prior to cell fixation. Data are presented as mean ± standard deviation of the mean. Ns, not significant (Kruskal-Wallis test with Dunn's multiple comparisons test).

**Figure S6. Lack of HSV-1 neutralizing antibodies results in efficient infection of neurons from HSV-1-infected ARPE-19 cells seeded in the NC, regardless of the presence of gG.** Neurons and mock- or HSV-1-infected ARPE-19 cells were seeded on the somal compartment (SC) and neurite compartment (NC) of MFC, respectively. No immunoglobulins were applied in the culture system. Cells were fixed at 24 hours post-seeding. Nuclei were stained with DAPI (blue), neurons were labelled with β-III-tubulin (TuJ1, green) and infected cells were detected by mCherry expression (red). Scale bar: 200 µm.

**Figure S7. Equal access to neurites in the NC results in similar neuronal infection with HSV1-CheGL and HSV1-CheGL-ΔgG.** (**A**) Immunofluorescence microscopy images of dissociated SCG neurons and infected ARPE-19 cells grown in MFC. Primary SCG neurons from neonatal mice were seeded on the somal compartment (SC) of the MFC. Infected ARPE-19 cells were seeded on the neurite compartment (NC) 24 hours later, when the neurites had grown through the microgrooves into the NC, in the presence of HSV-1 neutralizing antibodies. The cells were fixed at 24 hpi and labelled with anti-β-III-tubulin antibody (green) and stained with DAPI (blue). HSV-1 infected cells were detected by mCherry expression. Scale bar: 200 µm. (**B**) Graph showing the number of mCherry positive cells per image taken randomly on the SC. Each symbol represents one image. Data are presented as mean ± standard deviation of the mean. Unpaired t-test; ns, not significant.

**Figure S8. Purified HSV-1 virions do not affect neurite outgrowth. (A)** Cell-free virions were purified by ultracentrifugation through a 10% nycodenz cushion followed by sedimentation in a linear nycodenz gradient. The arrow points to the band that was collected for further analysis. **(B)** Immunoblot detecting HSV-1 gG and major capsid protein VP5 in purified HSV-1 virions. **(C)** Top panels show representative immunofluorescence confocal images of SCG incubated with neuronal medium or neuronal medium with purified HSV-1 virions. The media were treated with ultraviolet light (UV) to inactivate HSV-1. After 20-24 hours, SCG were fixed and labelled with anti-β-III-tubulin antibody (TuJ1, green) and stained with DAPI (blue). Scale bar: 200 μm. The graph below shows the quantification of neurite outgrowth in each experimental group, presented as the ratio of neurite fluorescence intensity (TuJ1) to DAPI signal intensity for each SCG. Quantification was performed with FIJI software (see Materials and Methods). Each symbol corresponds to one SCG. *P* values obtained by Kruskal-Wallis test with Dunn's multiple comparisons test are shown.

**Figure S9. Overexpression of galectin-1 increases the percentage of EVs containing galectin-1 at the surface.** VRFs derived from vector or galectin-1 transduced ARPE-19 cells were incubated with fluorescent-conjugated anti-galectin-1 antibodies at 4°C for 2 hours. EVs were washed through ultracentrifugation, resuspended in 100 µL PBS, and analyzed by FACS for galectin-1. Data were processed using FlowJo.

**Figure S10. Original immunoblot images.** The sections surrounded by red rectangles correspond to those shown in the figures of the manuscript.
