# Supplementary material for: Herpes simplex virus type 1 modifies the protein composition of extracellular vesicles to promote neurite outgrowth and neuroinfection
Source: mBio. 2024 Jan 26;15(2):e03308-23. doi: 10.1128/mbio.03308-23 (PMC10865794; doi:10.1128/mbio.03308-23)
Supplement: Supplemental Table Legend — Legend to Table S1. [file mbio.03308-23-s0004.docx]

**Table S1: Normalized quantitative protein data from mass spectrometry-based analyses.** To determine the protein composition of VRF obtained from vector- and HSV-1 gG-transduced ARPE-19 cells, liquid chromatography-mass spectrometry (LC-MS/MS) were performed. Raw data were processed using MaxQuant software with 1% FDR for peptide and protein identifications via internal Andromeda search engine to search MS2 spectra against a decoy human UniProt database (HUMAN.2019-07) and HSV Uniprot database (9294) containing forward and reverse sequences. Five replicates were assigned to each experimental group and data were filtered for minimum value of three in at least one group, and for proteins with a minimum of three MS/MS counts. Of the 214 proteins included for quantitation, 178 have been previously annotated as proteins present in the extracellular milieu and were listed in the table. Differential protein abundance was calculated using Student´s t-test and significantly regulated proteins were defined with a cut-off of 15% FDR. Raw data and full protein output tables are available via ProteomeXchange with identifier PXD039569.
